# Qualitative and Quantitative Analysis of a Novel Dental Health Education Program to Improve Dental Care Utilization by Bhutanese-, Burmese-, and Swahili-Speaking Refugees in a Midwestern United States City

**DOI:** 10.1155/ijod/8673757

**Published:** 2025-05-24

**Authors:** Chandra Swanson, Katherine Gordon, Kelly Kreisler, Elizabeth Montgomery Collins

**Affiliations:** ^1^Division of Pulmonary Medicine, Boston Children's Hospital, 300 Longwood Avenue, Boston, Massachusetts 02115, USA; ^2^Catholic Charities of Northeast Kansas, Kansas City, Kansas, USA; ^3^Health Partnership Clinic, Olathe, Kansas, USA; ^4^University of Global Health Equity, Kigali, Rwanda; ^5^Division of Hospital Medicine, Washington University School of Medicine in St. Louis, St. Louis Children's Hospital, St. Louis, Missouri, USA

**Keywords:** dental care utilization, immigrant and refugee health, oral health, oral hygiene, refugees

## Abstract

**Objectives:** Refugees have greater oral disease than native-born populations, yet underutilize dental healthcare resources. Few studies have evaluated the impact of health services and interventions seeking to improve dental care utilization among refugees, and dental healthcare utilization and outcome information for Bhutanese-, Burmese-, and Swahili-speaking refugees is sparse. This study aims to evaluate the impact of a novel culturally tailored education program on refugee dental knowledge, beliefs, behavior, and utilization in Wyandotte County, Kansas, United States.

**Methods:** Mixed methods, including discussion groups, World Café sessions, and structured pre-post intervention surveys, were used. Thematic analysis identified themes from qualitative data collected through community discussion groups (*n* = 14) and a World Café session (*n* = 22) with refugee community members. McNemar's chi-squared test and Wilcoxon signed-rank tests were used to evaluate changes in primary and secondary outcomes from quantitative data collected through preintervention surveys (*n* = 48) and postintervention surveys (*n* = 37).

**Results:** Pre-intervention, participants had dental knowledge and behaviors comparable to low-income Kansas populations. Postintervention, more participants knew correct brushing and check-up frequency, believed they lacked appropriate dental care, chose the dentist's office for their dental care, reported correct teeth brushing behavior, and completed dental visits than they did preintervention.

**Conclusions:** Providing dental education improved many parameters of dental refugee health; however, dental education alone did not increase all dental health knowledge, beliefs, behaviors, and utilization sufficiently. Rather, using the World Café methodology for refugee patient feedback, and assisting refugees with dental healthcare accessibility, affordability, and accommodation during insurance registration and dental clinic attendance may more effectively improve oral healthcare utilization and dental health outcomes in refugee populations.

## 1. Introduction

Oral diseases represent a public health concern and negatively impact general health and quality of life; the burden of oral disease falls disproportionally on disadvantaged child and adult populations [1, 2]. While literature evaluating refugee dental health is sparse, particularly regarding Burmese, Nepali-speaking Bhutanese, and Swahili-speaking populations, refugees have higher rates of oral disease compared to native populations and other immigrants in resettlement countries [3–6]. Clinical examinations of Syrian refugees in Canada revealed poor oral health, with many seeking emergency care only [4]. Often, refugees require care beyond routine cleaning at their first visit [5]. A study comparing diagnoses in refugee patients to native-born patients in the United States (US) found female refugees had a 2.1 higher relative risk for teeth and jaw disorders in emergency visits and male refugees a 7.9 higher relative risk of teeth and jaw disorders in outpatient settings [7]. Several factors contribute to dental disparities. Studies identified detrimental cultural dietary and oral hygiene practices, including that Burmese and Karen families often consume excess juice and sugared beverages and traditionally clean teeth with charcoal [8, 9]. Lack of access to dental services in the country of origin often occurs due to discrimination, transitory lifestyle, and/or lack of infrastructure. Despite the increased disease burden, non-US citizens and naturalized citizens have lower dental service utilization than US-born citizens [10]. Barriers facing non-US citizens in utilizing dental healthcare include inaccessibility, cost, and lack of insurance [1]. However, Australian studies revealed even when no-cost dental services were available to migrants, low rates of utilization still occurred due to confusion about which services were free [11, 12]. Additional reasons for service underutilization by Somali, Afghani, and Sri Lankan communities in the United States and Australia include reliance on emergent care, fear of care (particularly during pregnancy), degree of acculturation, and low health literacy [12, 13].

Publications often conclude that refugees and migrants benefit from oral hygiene education to improve knowledge about dental health [1, 3, 5, 12, 14, 15]. Outreach to refugee populations to improve dental healthcare utilization is one way to promote health, yet few studies have evaluated the impact of health services and interventions seeking to improve dental care utilization among refugees [15, 16]. Models for achieving primary healthcare delivery have included coordinating service networks (accessibility), interventions increasing awareness and health literacy through media and health education (availability), reducing costs (affordability), and use of interpreters, bilingual workers, and outreach programs to facilitate registration and clinic attendance (accommodation) [14, 17, 18].

Major refugee ethnicities represented in Wyandotte County, Kansas, include Chin, Karen, Karenni, and Burmese from Burma (30%), Lhotshampa from Bhutan/Nepal (10%), and various ethnic groups (including Swahili-speaking) from the Democratic Republic of Congo [19]. As seen nationally, refugees in Kansas have extensive oral health needs and underutilize dental services; from October 2017 to January 2018 and in 2020, the Kansas Office of Refugees reported that dental issues were among the top diagnoses during initial domestic refugee medical screening exams [20]. Wyandotte County dental services are limited—inaccessible/unaffordable dental care was identified as a problem in half the county zip codes [21]; there is only one dentist per 3,019 individuals (below the Kansas and national averages), and only 24.3% of Kansas dentists participate in Medicaid (below the national average of 38.6%) [22]. In 2016, Vibrant Health Dental Clinic (VHDC) created an adult refugee clinic in Wyandotte County. Though the Centers for Disease Control and Prevention (CDC) recommends all refugees receive an oral health examination [23], VHDC records indicate many refugees miss their dental appointments. Refugee patients seen at VHDC also lack dental insurance; 18% of refugee patients had public or private insurance, while 55.0% of all adults in the region had private dental coverage [24].

This project aimed to (1) evaluate perceptions of dental healthcare by refugees in Wyandotte County, Kansas, United States, (2) create culturally tailored educational materials addressing identified barriers to utilization, and (3) improve dental health outcomes following participation in this novel refugee community needs-based dental health education program. We hypothesize that participation in the novel refugee community needs-based dental health education program will improve dental healthcare utilization, knowledge, beliefs, and behaviors among the sampled population.

## 2. Methods

This study was performed within the principles of the Declaration of Helsinki. Approval was granted by the University of Kansas Medical Center Human Subjects Committee (Improving Dental Care Utilization by Refugee Patients in Wyandotte County, Kansas; July 3, 2017, STUDY00143499).

### 2.1. Participants and Data Collection

The data collection methodology is summarized in Table 1.

To create the novel and targeted educational materials, one researcher (CS) first completed unstructured, participant observation of refugee health assessments as a community health intern at Wyandotte County Refugee Medical Home clinics. Next, convenience sampling was used to recruit Wyandotte County Community Health Council community health workers (CHWs) (*n* = 7) who were all established refugees in the community and Catholic Charities Refugee Office staff (*n* = 7) for focused, structured interviews from June to July 2017. A working relationship with the refugee population and these organizations had been established prior to this study. Four educational handouts (translated into Nepali and Burmese) and four videos (four interpreted into Nepali and three into Burmese) were created to address barriers identified during the interviews to refugees' dental healthcare utilization (Supporting Information: Appendix 1). To improve these original educational videos and handout materials, two semistructured community discussion groups with the CHWs and case workers and one World Café session at Catholic Charities with Bhutanese and Burmese adult refugee community members (*n* = 22) were conducted from August 2017 to January 2018.

The World Café data collection method is a small-group or round-table format designed to create a collaborative and hospitable environment for gathering diverse individual and collective knowledge; its use within refugee communities has been previously described [25]. It is one of several best practices this study employed to ask our research questions of our research subjects, while addressing the unique ethical challenges common to refugee research [26]. The World Café method allows researchers to approach ethical challenges by focusing on values (such as communal decision-making) of target communities, respecting individual privacy (not requiring individual responses), and providing an outlet for community feedback prior to finalizing the educational materials to improve cultural appropriateness (competence/sensitivity).

Snowball sampling was used to recruit pre- and post-survey participants [27, 28]. Power and sample size calculations were performed with a statistician; however, a convenience sample of as many participants as possible from the community refugee resettlement agency was utilized. Eligibility criteria included self-identification as a refugee community member and age greater than 18 years old. Discussions were facilitated by two researchers (KK, CS) in English with Nepali and Burmese interpretation by three experienced refugee community members. No formal relationship between the researchers and refugee community members was established prior to study initiation; researcher roles and research goals were presented at each session. Interpreters recorded discussion highlights, and facilitators added notations. Educational materials were iteratively improved utilizing this feedback; for example, participants recommended ways to better communicate key points to improve their comprehension and recommended recruiting refugee community members for photographs during their dental health appointments to provide culturally tailored images (Supporting Information: Appendix 1).

To evaluate if educational materials improved participant dental health knowledge, attitudes, behaviors, and utilization, Burmese-, Bhutanese-, and Swahili-speaking refugee community members were recruited by self-selection sampling after viewing fliers distributed in Catholic Charities citizenship classes. Participating refugees completed a pre-survey and dental educational session February–March 2019 (*n* = 48); of those participants, 77% completed a post-survey (*n* = 37) 6 months later, August–September 2019, in person or via telephone. A piloted survey was improved based on interpreter feedback. Surveys (Supporting Information: Appendix 2) were administered by the research team and experienced community members in English or interpreted into Nepali, Burmese, or Swahili. Surveys were interpreted rather than translated since community feedback indicated many refugees were not able to read in their primary spoken language. During the educational session, participants selected English, Nepali, or Burmese handouts and accompanying videos to review or had them interpreted into Swahili. Participants received lunch during their educational session and a $10 gift card after completing the pre-survey and another following the post-survey.

### 2.2. Qualitative Analysis

Qualitative, deductive, and inductive thematic analysis was conducted using approaches described by Braun and Clarke and further informed by the approach of Roberts, Dowel, and Nie [29, 30]. First, discussion transcripts were compiled and read in detail to ensure familiarity with the data. Next, recurring words or ideas were highlighted, with each assigned a unique color. This was completed manually in Microsoft Word (Version Microsoft Office Standard 2016, Microsoft Corporation, Redmond, Washington). The initial codes were then discussed, clarified, and refined. For instance, the infrequently occurring codes of tobacco, alcohol, and diet that arose from community focus groups were combined into the code “substances that affect dental health” (Table 2). Codes were next organized into themes, with particular attention to relevance to our project aims. These themes were reviewed to ensure each was coherent, distinct, and supported by the coded data. Finally, each theme was placed into a category of the socioecological model (SEM).

The SEM is a theoretical framework that assumes an individual's behavior is a result of a dynamic interaction of intrapersonal characteristics, interpersonal processes, institutional factors, community features, and public policy [31]. Past studies have applied SEMs to examine barriers to mothers improving the health or oral health of their children in minority, underresourced, and immigrant populations [32, 33], and supported the use of SEMs to identify barriers to dental healthcare in populations [34]. Thus, the SEM framework was used to categorize dental health themes identified during community focus groups and reveal interventions that would encourage or prevent refugees from engagement with dental healthcare systems.

Code and theme identification were conducted by the primary author and affirmed by the research team with training and experience in qualitative research. All reviewers were female, from the United States, working within an academic institution in the Midwest, and had prior experience working with refugee populations. Three of the authors were residents of the Kansas City metropolitan area while the study was conducted. The primary author was a medical student, second author a law student, and remaining authors pediatricians at the time of the study. All authors considered their underlying biases, prior assumptions, racial and immigrant mix, and prior experiences when analyzing participants' statements. The Consolidated Criteria for Reporting Qualitative Research Checklist was completed for manuscript submission [35].

### 2.3. Quantitative Analysis

The survey included questions on demographics, primary outcomes (dental healthcare utilization), and secondary outcomes (knowledge, beliefs, and behavior). Survey data was managed using REDCap hosted at the University of Kansas Medical Center [36]; participants lost to follow-up (*n* = 11) were excluded from the analysis. Microsoft Excel (versions 1908–2002, Microsoft Corporation, Redmond, Washington) was used to summarize data and perform statistical calculations. Absolute and relative frequencies were calculated for demographic variables and outcomes of interest. RStudio was used for statistical analysis [37]. Nonparametric statistical tests including McNemar's chi-squared test with continuity corrections [38], binomial test for small sample sizes, and the Wilcoxon signed-rank test for continuous variables [39] were used to evaluate changes in primary and secondary outcomes. *P*-values were deemed statistically significant when *p*-values were less than 0.05. A statistician reviewed the design, methods, results, and content of this paper.

## 3. Results

**Figure 1:**
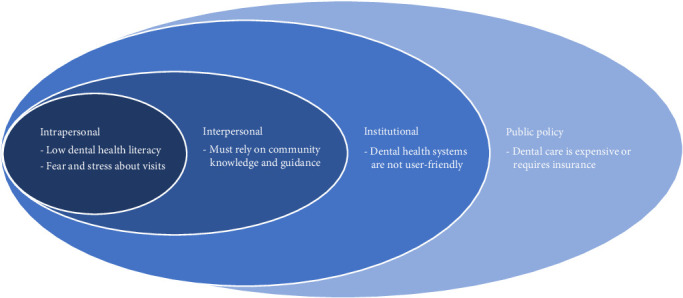
Socioecological model for refugee dental health utilization: four tiers and five themes.

Community discussion groups and the World Café session revealed codes that fell into four socioecological tiers: (1) intrapersonal, (2) interpersonal, (3) institutional, and (4) public policy; community themes were identified in the interpersonal tier. Within those tiers, five themes regarding barriers to refugee dental healthcare emerged: (1) refugees have low dental health literacy, (2) refugees have fear and stress about dental visits, (3) refugees must rely on community knowledge and guidance, (4) dental health systems are not user-friendly, and (5) dental care is expensive or requires insurance (Figure 1 and Table 2).

Specific demographic characteristics of World Café participants were not collected in order to preserve anonymity and encourage participants to speak freely. Eight World Café participants were Burmese-speaking, and 14 were Bhutanese-speaking. Demographic characteristics of pre- and post-survey respondents are presented in Table 3. The uninsured status in the pre- and post-survey groups ranged from 39.4% to 61.1% (potentially up to a maximum of 69.4% if patients with missing data were uninsured).

Following the educational sessions, the percentage of participants that completed a dental visit trended upwards from 42.4% to 60.6% (*p*=0.18) but did not reach statistical significance. Discordantly, there was a statistically significant increase in time since last dental visit from 4.7 months to 14.8 months. Given the time between pre- and post-surveys was 7 months, this increase is likely spurious. The percentage of participants who had scheduled an appointment trended downwards from 41.2% to 32.4% (*p*=0.82), and the time since last scheduled appointment trended upwards from 1.5 to 2.5 months (*p*=0.58) (Table 4), but these results did not reach statistical significance.

Table 5 summarizes secondary outcomes following the educational sessions: changes in dental health knowledge, beliefs about the quality of care received and importance of dental health, and behavior regarding flossing, brushing, and selecting a dentist. While there were no statistically significant differences between pre-survey and post-survey secondary outcomes, positive trends were seen in brushing knowledge and behavior, dental visit knowledge and behavior, and dental health beliefs.

## 4. Discussion

In this study, we successfully evaluate Bhutanese-, Burmese-, and Swahili-speaking refugee knowledge, behaviors, and dental service utilization and reveal unique aspects of the participant refugee experience affecting dental health-seeking behavior that can serve as guideposts for implementing change. We detail the creation of a culturally tailored educational intervention, guided by refugee community feedback, to address commonly identified barriers to optimal dental health. While tailored educational handouts and videos did improve almost all areas of refugee participants' measured dental knowledge, beliefs, behaviors, and service utilization, none of the improvements were statistically significant, most likely due to the small sample size. Rather, we found that refugee dental health outcomes may be most improved via a combined approach of dental health education and interventions that target systemic barriers such as cost of care.

The effectiveness of oral health educational programs with other refugee and migrant populations has been mixed. Refugee patients in a tailored interprofessional educational program had improved oral health literacy [40], and migrant farmworkers demonstrated successful retention of oral health knowledge after a CHW-led oral health knowledge session [41]. Conversely, an oral health educational program directed at refugee patients living in the United States for less than a year, including Burmese- and Nepali-speaking members, did not change oral health-related knowledge, attitudes, or behaviors [42]. Our study supports, as these study authors suggest, that rather than education alone, multiple interventions in combination with an educational program are needed to significantly improve refugee oral health knowledge, attitudes, and dental healthcare utilization.

Intrapersonally, many refugees have low dental health literacy, limiting the understanding of oral health information and systems in their home and host countries. Per the World Café discussion, though many participants consider their oral health important, they had low awareness of available standard dental services. After the session, more participants understood they lacked appropriate dental care, identified the dentist's office as the most appropriate location for dental services, and completed dental visits.

As prior research has shown, we found that lack of knowledge can also contribute to stress and fear that refugees associate with dental care [13] (Table 2). Often, dental manipulation causes discomfort and increases teeth sensitivity, which participants reported interferes with eating preferred foods and working (Table 2). By providing education targeting cultural misconceptions and removing unknowns about dental visits, we improved acceptability and planned utilization of dental healthcare—the number of participants who believed they were not receiving appropriate dental care, believed dental health was of equal or greater importance than overall health, and would go to a dentist office for dental healthcare all increased. Flossing was the only knowledge and behavior category that did not improve, perhaps because it was unfamiliar and can increase pain, bleeding, and cost. Targeted education may be required to teach flossing and reassure refugees that pain and bleeding decrease with routine flossing. However, since participants understood brushing, flossing, and dental visit frequency recommendations at the same level as average low-income Kansas residents, and when combining reported home brushing and flossing behaviors, 80.8% of our participants practiced appropriate behaviors before and 86.6% after the session [43], improvements in knowledge and behaviors alone may not increase refugee oral healthcare utilization.

Institutionally, dental check-ups can be difficult to navigate. Refugee patients are often referred to dentists via primary care physicians or present to other locations seeking dental healthcare which complicates access to and increases time until dental services. Appointments are often scheduled via phone or computer, where language barriers can impede access. Participants were more likely to see the dentist for acute symptoms than for preventive check-ups. Successful urgent care visits must promote continuity of care, which can encourage others in refugee communities to seek dental healthcare at dentists' offices. Interventions tailored to refugee community needs, such as increasing numbers of same-day appointments and providing interpreters for phone scheduling and visits, could improve the availability, accessibility, and utilization of dental health services. From the dental provider's perspective, additional areas of preventive dental care could be emphasized. Historically, fluoride has been the first-line treatment used for preventive purposes in dental practice [44]. Recently, casein phosphopeptide-amorphous calcium phosphate and biomimetic hydroxyapatite have been introduced and showed promising results [45, 46]. These preventive strategies could be utilized to reduce caries risk.

Public policy barriers to dental healthcare utilization include lack of insurance and high cost—the most common concern heard from participants (mentioned 41 times; Table 2). CHWs described instances where cost limited the care continuity and forced them to recommend alternatives to standard primary care dental services. CHWs and providers should emphasize that the health consequences of foregoing dental check-ups often outweigh the costs of the check-up. Improving affordability can promote continued dental service utilization; however, without improved refugee dental insurance infrastructure, providers have limited ability to provide long-term, consistent care to refugees.

Both pre- and post-survey groups reported lower dental insurance coverage (30.6%–48.5%) than the Kansas average of 64% [24]. Many respondents were uninsured; however, dental insurance information was not collected from all respondents. Regardless, in nonrefugee populations, lack of insurance decreases dental visit frequency [47]. We conclude that in refugee populations, increasing insurance coverage is necessary to improve dental health utilization [1]. Solely expanding baseline Medicaid coverage may not fully meet our study population's needs because most refugees require more extensive cleaning and care than had been provided in initial assessments. Though this has improved in Kansas since 2019 due to advocacy efforts, continued advocacy is needed for insurance companies and states to provide appropriate dental services coverage for refugees. These barriers are consistent with a recent systematic review that identified affordability, awareness, and accommodation as the most common barriers to dental care for refugees in highly developed countries [48].

## 5. Limitations

This study had limitations. For survey respondents, the median length of time in the United States was 48 months, so our population differs from newly arrived refugees. As identified in other literature, because our participants had been in the United States longer and had a greater amount of time for engagement in medical services and acculturation than new arrivals, our participants may have been less impacted by educational materials than new arrivals would have been [13]; therefore, our study underestimates the impact of our materials on new arrivals. We recommend more research with newer arrivals to discover if more impact can be made on dental healthcare utilization.

The only statistically significant difference in pre- versus post-data was the data point describing the months since the last dental visit. However, the reported time since the last visit increased from 4.7 to 14.8 months, which would reflect worse dental healthcare utilization. Since a maximum of 7 months had passed between pre- and post-survey dates, we suspect the reported increase is due to recall or participant bias, and we deemed the answers to that survey question inaccurate.

The sample size of this study is also small and likely underpowered. To better evince the statistical significance of the other positive trends, future studies should focus on a larger sample size. Furthermore, language and literacy barriers can impede the impact of videos and written handouts in educational sessions. While translation of materials into Nepali and Burmese allowed outreach to a large portion of our target populations, many refugees from Burma speak languages other than Burmese. Multiple translations were prohibitively time-consuming or expensive for our budget. Once, it was observed that some participants answered survey questions as a group; anecdotally, this pattern of group answering has been seen in Bhutanese refugees. Additionally, we observed social desirability bias with participants turning to interpreters for correct answers and that participants did not answer all questions, impeding our ability to interpret some data, particularly data measuring time. Thus, refugee research surveys and their instructions should be reviewed in detail with interpreters before administration. Intentionally designed to be collaborative and nonprescriptive, World Café methodologies were more effective than surveys in gathering accurate *individual* responses; we recommend this method and intensive translation and interpretation assistance be employed in refugee research to ensure all survey questions are answered accurately.

Last, 22.9% of pre-survey participants were lost to follow-up due to refusal (*n* = 3), nonworking telephone numbers (*n* = 4), and unknown reasons (*n* = 4). Those lost to follow-up were more likely to have completed pre-surveys in March, be older, and speak Nepali. Future investigations could determine if those groups need specific follow-up interventions.

The transferability of our qualitative results and external validity of our quantitative results are limited as participants were recruited via nonprobability sampling and were offered a lunch and $10 gift card incentive for participation. Ascertainment bias may limit generalizability; we attempted to limit bias by administering surveys on multiple days/times to accommodate a wide pool of participants. Additionally, participants represented only three language groups and were resettled by one agency to one geographic location. Finally, to preserve the tenet of individual privacy emphasized in World Café methodology, specific demographic information on World Café participants was not collected; this could limit transferability.

Our findings should be extrapolated with careful consideration to refugees from other countries of origin and their unique exposures to dental healthcare as well as the regional health insurance and policy context of their resettlement communities. However, the strategies and approaches described (World Cafe, targeted written and multimedia education) may prove effective in improving oral health across a variety of refugee communities.

## 6. Conclusions

With the limitations described, this study provides insights into multilevel socioeconomic factors encouraging and limiting the utilization of dental healthcare by refugees, especially Bhutanese-, Burmese-, and Swahili-speaking refugees. This is the first time the World Café tool was reported to be used to gather community member perspectives to tailor dental educational materials; this tool may have applications beyond refugees and dental healthcare. Novel educational materials were developed by the authors with refugee community members and shared here to address community-identified barriers and promote dental health and healthcare utilization. This study showed that education alone did not remove oral health disparities between refugees and native populations. Rather, additional measures to enhance dental health knowledge, change dental health beliefs and behaviors, and increase dental health utilization by refugee populations may be needed. These include targeted clinic hours, increased availability of interpreters, lowered costs, and increased insurance coverage. Further studies are needed to better understand and evaluate the impact of these and other educational materials and to determine best practices for improving refugee dental healthcare utilization and health.

## Figures and Tables

**Table 1:** Summary of data collection methodology.

Method	Dates	Sampling technique	Number of participants
Structured interviews	June–July 2017	Convenience sampling	14
Community discussion groups	August 2017–January 2018	Convenience sampling	14
World Café	January 2018	Convenience sampling	22
Pre-survey	February–March 2019	Snowball sampling	48
Post-survey	August–September 2019	Snowball sampling	37

**Table 2:** Frequency of topics regarding barriers to and promotion of dental care access for refugee populations identified in discussion groups.

SEM^a^ model tiers	SEM model themes	SEM model codes	Sample quotes	CHW^b^ (*n* = 7)	Case workers (*n* = 7)	Refugees (*n* = 22)	Total (*n* = 36)
Intrapersonal	Low dental health literacy	Pain motivating care-seeking behavior	“Someone should go to the dentist to get relief from toothache”	8	2	5	15
		Tobacco/alcohol/diet/substances that affect dental health	“Tobacco and betel nut are very common in our community. It's very important to know they are bad” “We learned especially about snacking”.	2	5	21	28
		Education/information	“Don't know what floss is” “Don't know why they have to go”	22	1	0	23
	Refugees have fear and stress about dental visits	Fear/stress	“Scared of side affects after clean up” “If I go to the dentist, I will be shaky and can't go to work” “Hard to eat the things we like after visit”	13	1	1	15

Interpersonal	Reliance on community knowledge and guidance	Community knowledge	“Use cloves for tooth pain” “If it works for one person, word spreads quickly”	16	0	13	29

Institutional	Dental healthcare systems are not user-friendly	Structural/institutional barriers	“Teach us how to schedule” “Have to have a referral”	11	6	6	23

Public policy	Expensive or requires insurance	Expense	“You need money to have dental health”	25	2	14	41

	—	Total by group		97	17	60	174

^a^SEM refers to socioecological model.

^b^CHW refers to community health worker.

**Table 3:** Respondent demographics for pre- and post-surveys^a^.

	Pre-survey Feb–Mar 2019 (*n* = 48)	Post-survey Aug–Sept 2019 (*n* = 37)
Average age (standard deviation)	38.0 (12.5)	37.1 (9.3)
Gender^b^		
Female	8 (16.7%)	7 (18.9%)
Male	12 (25.0%)	10 (27.0%)
Unknown	28 (58.3%)	20 (54.1%)
Preferred language (self-reported)^c^	(*n* = 47)	(*n* = 37)
Burmese-/Myanmar-speaking	14 (29.8%)	11 (29.7%)
Myanmar	4 (8.5%)	0 (0%)
Burmese and Zo Tung Chin	1 (2.1%)	0 (0%)
Burmese and Lautu Chin	0 (0%)	3 (8.1%)
Burmese, Lautu Chin, and Falam Chin	0 (0%)	1 (2.9%)
Hakka Chin	0 (0%)	1 (2.9%)
Lautu Chin	7 (14.9%)	4 (10.8%)
Zotung Chin	2 (4.3%)	2 (5.4%)
English	0 (0%)	2 (5.4%)
Kinyarwanda	7 (14.9%)	4 (10.8%)
Kirundi	1 (2.1%)	0 (0%)
Nepali	13 (27.7%)	8 (21.6%)
Swahili-speaking	12 (25.5%)	12 (32.4%)
Swahili	11 (23.4%)	10 (27.0%)
Swahili and English	1 (2.1%)	1 (2.9%)
Swahili and Kirundi	0 (0%)	1 (2.9%)
Average months in United States (standard deviation)	(*n* = 47) 44.6 (29.8)	(*n* = 37) 47.2 (27.5)
Dental health insurance status	(*n* = 33)	(*n* = 36)
Have dental insurance	16 (48.5%)	11 (30.6%)
Do not have dental insurance	13 (39.4%)	22 (61.1%)
Status not known	4 (12.1%)	3 (8.3%)

^a^Some participants were lost to follow-up and did not take the post-survey (*n* = 11). The *n* listed in the table reflects the number of respondents for each question; not all participants responded to each question.

^b^Gender data was not self-reported on surveys; gender was reported retrospectively by the refugee community members/translators who categorized participant names as male, female, or unknown.

^c^Myanmar and Burmese are considered the same language. In this paper, “Burmese” refers to refugees from Burma who speak Burmese and are primarily members of the Chin, Karen, or Karenni ethnic groups; “Nepali-speaking Bhutanese” or “Bhutanese” refers to refugees from Bhutan (by way of Nepal) who speak Nepali and are primarily members of the Lhotshampa ethnic group, and “Swahili-speaking refugees” refers mainly to refugees from the Democratic Republic of Congo who speak Swahili and represent a variety of ethnic groups.

**Table 4:** Primary outcomes: comparison of dental healthcare utilization in matched pairs of pre–postsurvey respondents.

	*n* = number of matched respondents that answered question	Pre-survey Feb–Mar 2019	Post-survey Aug–Sept 2019	*p*-Value
Have ever visited the dentist (*n*, %)	33	14 (42.4%)	20 (60.6%)	0.18^a^
Average number of months since last dental visit (*n*, standard deviation)	13	4.7 (8.4)	14.8 (18.1)	0.02^b^
Have ever scheduled a dental visit for self (*n*, %)	34	14 (41.2%)	11 (32.4%)	0.82^a^
Average number of months since last scheduled dental visit for self (*n*, standard deviation)	2	1.5 (2.9)	2.5 (2.8)	1.0^b^

^a^The *p*-values were calculated using McNemar's chi-squared test in R Studio.

^b^The *p*-value was calculated using the Wilcoxon signed-rank test in R Studio.

**Table 5:** Secondary outcomes: knowledge, beliefs, and behaviors regarding dental healthcare in matched pairs of pre–post survey respondents.

	*n* = number of matched respondents that answered question	Pre-survey Feb–Mar 2019	Post-survey Aug–Sept 2019	*p*-Value
Know that correct brushing frequency is at least twice daily (*n*, %)	33	31 (93.9%)	32 (97.0%)	0.97^a^
Know that correct flossing frequency is at least once daily (*n*, %)	33	26 (78.8%)	25 (75.8%)	1^b^
Know that correct dental visit frequency is twice a year (*n*, %)	33	13 (39.4%)	14 (42.4%)	0.57^b^
Believe they do not receive appropriate care (*n*, %)	35	17 (48.6%)	26 (74.3%)	0.07^b^
Believe that dental health is of equal or greater importance than overall health (*n*, %)	27	26 (96.3%)	27 (100.0%)	1^b^
Brush at least twice a day (*n*, %)	26	20 (76.9%)	24 (92.3%)	0.13^b^
Floss at least once a day (*n*, %)	26	22 (84.6%)	21 (80.8%)	1^b^
Choose the dentist/dentist office as preferred place to go when having problems with teeth (*n*, %)	26	14 (53.8%)	21 (80.8%)	0.10^b^

^a^The exact *p*-value was calculated with the binominal test in R Studio. Using the standard that a *p*-value < 0.05 represents statistical significance, no statistically significant differences were found in secondary outcomes.

^b^The *p*-values were calculated using McNemar's chi-squared test with continuity correction in R Studio; no *p*-values met statistical significance of less than 0.05.

## Data Availability

The datasets generated during and/or analyzed during the current study are available from the corresponding author upon reasonable request.

## Supporting Information

Supporting Information 1 Appendix 1 includes the participant feedback used to refine the initial educational materials, links to the educational videos, images of the English language educational handouts, and links to the online educational handouts the authors created in a variety of languages. The authors received an American Academy of Pediatrics (AAP) Kansas chapter grant to translate educational materials into more languages with wider dissemination through Catholic Charities and other community health organizations; a single-page summary handout has been created and translated into Spanish, Swahili, Burmese, Nepali, Pashto, Somali, and Arabic. These handouts are available in the Supporting Information or by contacting the corresponding author. Appendix 2 includes the final survey instrument.

Supporting Information 2 The Consolidated criteria for Reporting Qualitative Research Checklist was completed for manuscript submission and can be reviewed in the Supporting Information.
