# Supplementary material for: Qualitative and Quantitative Analysis of a Novel Dental Health Education Program to Improve Dental Care Utilization by Bhutanese-, Burmese-, and Swahili-Speaking Refugees in a Midwestern United States City
Source: Int J Dent. 2025 May 24;2025:8673757. doi: 10.1155/ijod/8673757 (PMC12126264; doi:10.1155/ijod/8673757)
Supplement: Supporting Information 1 — Appendix 1 includes the participant feedback used to refine the initial educational materials, links to the educational videos, images of the English language educational handouts, and links to the online educational handouts the authors created in a variety of languages. The authors received an American Academy of Pediatrics (AAP) Kansas chapter grant to translate educational materials into more languages with wider dissemination through Catholic Charities and other community health organizations; a single-page summary handout has been created and translated into Spanish, Swahili, Burmese, Nepali, Pashto, Somali, and Arabic. These handouts are available in the Supporting Information or by contacting the corresponding author. Appendix 2 includes the final survey instrument. [file 8673757.f1.pdf]

Appendices

Appendix 1: Educational materials created to address identified barriers to refugee dental health utilization.

a. Participant feedback from iterative process for refining educational materials. All handouts were discussed together and main points were shared. Videos were not discussed at the World Café.

	Comments to researchers by participants			
	Community Health Council of Wyandotte County CHW and Catholic Charities Staff Feedback – August 2017	*Catholic Charities CHWs Feedback– October 2017*	*Vibrant Health Dental Staff Feedback – November 2017*	*World Café with Bhutanese and Burmese Refugee Community Members Feedback –January 2018[a]*
Dental Check-ups: When, Why, How	• Translate into multiple languages<br>• Focus on the consequences of not doing preventative care<br>• Add pictures of what happens when [patients] don't care for [their] teeth<br>• Collaborate with Catholic Charities staff members<br>• Add personal experiences from community members<br>• Let [patients] know when something (they believe) is incorrect<br>• Educate little by little<br>• Would be good to show in waiting rooms	• Add images of paycheck stubs, tax returns, unemployment letters, and describe them<br>• Remind them that clinic staff can help call a Medicaid taxi	• Add discussion of what the patient should do if they had an immediate issue<br>• Describe the phone tree, especially what to do if urgent dental issue arises<br>• We really care about avoiding snacking	• All the information on the fliers is very useful<br>• Tobacco and betel nut are bad<br>• Bad dental health has a bad effect on overall health<br>• We learned a lot, especially about snacking<br>• Healthy teeth are a worthwhile investment<br>• Money and teeth are associated<br>• Find a way to communicate with the young people

	• Make sure materials are simple, easy to understand<br>• Explain that tools [dental instruments] are not to sting but to clean<br>• Include a CHW or social worker to explain the video<br>• Explain that 1st and 2nd appointments are for diagnosing<br>• Make "KanCare" logo and images bigger on insurance card<br>• Explain that dentists are not going to fix everything at once			
What to Expect at your First Dental Visit	• Use more plain language<br>• Explain the word "referral"<br>• Bags of money image is unclear (does not clarify the cost-benefit of preventive dental care)<br>• Reorganize the explanation of how dental check-ups lead to good overall health<br>• Add proper way to brush teeth	• Remind viewers that this is an initial visit<br>• Visuals for dental tools are scary<br>• Emphasize that if (patients) are uncomfortable that they can say something to the dentist or dental assistant<br>• Explain why x-rays are done<br>• Explain fluoride<br>• Mention that x-ray vests are heavy	• Substitute the word "plaque" with "bacteria" or "germs"<br>• Our clinic uses gel or varnish instead of fluoride foam<br>• Check the translation for describing the lead apron<br>• Add a discussion of what the patient should do if they had an immediate issue	

Dental Health at Home	• Show refugees healthy habits for dental care<br>• Show both sides – what happens when you do and don't take care of your teeth<br>• Have repeated education sessions<br>• Add where to get floss, toothbrush<br>• Use real food for all pictures<br>• Add picture of chewing gum/bubble gum<br>• Write "yes" under good/correct images and "no" under bad/incorrect images<br>• Show picture and video of flossing	• Define "paan" [betel leaf/areca nut chewed as stimulant] for dentists<br>• Use more culturally relevant tobacco images	• Emphasize no snacking
Transportation/ How to Schedule an Appointment	• Remove arrows from under the footstep images<br>• Teach patients how to use the RideKC phone "app"<br>• Replace the open mouth picture with an image of beautiful teeth	• Fix embedded videos<br>• Remind patients to ask for an interpreter at the front desk	• No comments

b. Link to educational videos: https://www.youtube.com/playlist?list=PL_GTXX0d6hEIIV81K-IZf_iFJ1zTz4XMp.

c. Most recent version of educational handouts (shown here in English)

DENTAL CHECKUPS:
WHY, WHAT, WHEN, AND HOW

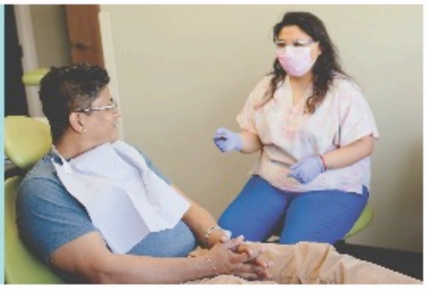

GOING TO THE DENTIST TWICE A YEAR KEEPS YOUR SMILE BRIGHT AND HEALTHY AND PREVENTS COSTLY HEALTH PROBLEMS.

DENTISTS HELP PROMOTE

- HEALTHY TEETH & BONES
- NUTRITIONAL HEALTH
- HEALTHY HEART

DENTISTS HELP PREVENT

- BAD BREATH
- TOOTH LOSS
- GUM DISEASE

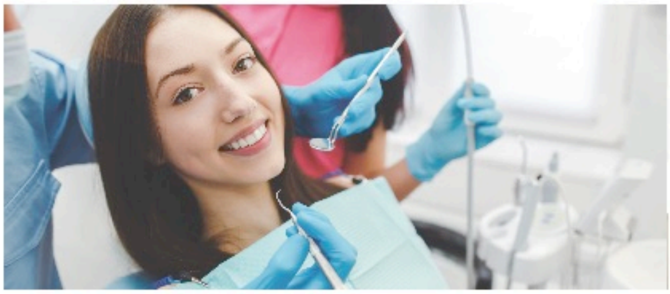

ESTIMATED COST

Cost depends on insuranoe type or household inoome. Vibrant Health oan oreate a personal payment schedule for you. FOR MORE INFORMATION CALL (913) 342-2552 THEN DIAL 2

WHAT TO EXPECT AT YOUR FIRST DENTAL VISIT

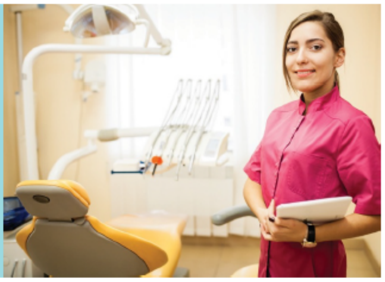

WHO WILL YOU SEE AT YOUR VISIT?

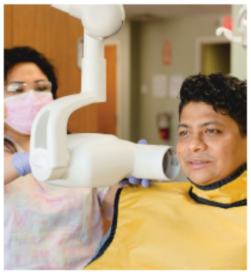

The office receptionist, then the dental hygienist and the dentist will see you.

WHAT WILL HAPPEN AT YOUR VISIT?

The dental hygienist will talk with you about your teeth and mouth (oral health) and general health.

X-RAYS

Pictures of your teeth and jaw will be taken while you are standing and seated.

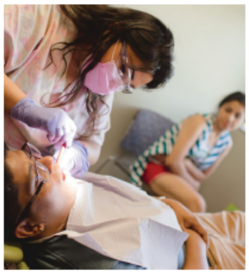

CLEANING AND FLUORIDE

The hygienist will brush and floss your teeth, gently remove sticky plaque and bacteria with special tools, ask you to rinse your mouth with water, and may coveryour teeth in a protective gel.

FIX AND FOLLOW-UP

The dentist will examine your teeth and fix any serious or urgent problems. the staff will discuss dental care at home and schedule your next appointment.

CONTACT VIBRANT HEALTH DENTAL CLINIC FOR MORE INFORMATION AT (913) 342-2552 THEN DIAL 2

WHEN SHOULD YOU GO TO THE DENTIST?

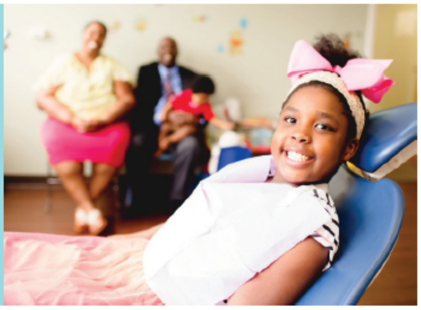

ANYTIME YOU HAVE AN URGENT DENTAL PROBLEM

FOR A DENTAL CHECK-UP EVERY 6 MONTHS

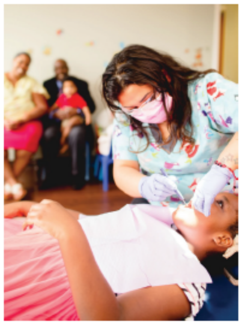

HOW TO SCHEDULE A DENTAL APPOINTMENT

1. CALL (913) 342-2552 THEN DIAL 2
2. TELL SCHEDULER IF YOU PREFER ANY CERTAIN DAY OFTHE WEEK, DATE, OR TIME FOR YOUR APPOINTMENT (INITIAL APPOINTMENTS LAST ABOUT 1 HOUR)
3. HAVE YOUR CALENDAR AND ANY INSURANCE CARDSOR DOCUMENTS IN HAND DURING THE CALL

PLEASE ARRIVE 20 MINUTES BEFORE YOUR APPOINTMENT TIME. WE LOOK FORWARD TO YOUR VISIT!

DENTAL HEALTH AT HOME

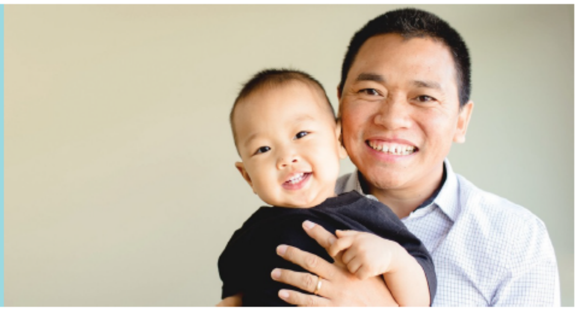

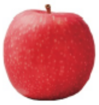

EAT HEALTHY FOOD

Avoid sugary foods and drinks. sugar feeds germs and creates acid that harms your teeth.

Crisp vegetables and fruits (like fresh carrots or apples) and calcium-rich foods(like almonds, milk or yogurt) will keep your teeth healthy and strong.

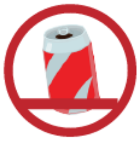

AVOID SNACKING

After eating or snacking, food and germs mix to make acid that attacks teeth.

Snacking without brushing your teeth increases food and acid left on teeth.

3 meals a day and fresh fruit or vegetable snacks, followed by brushing, reduces acid.

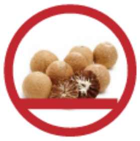

PREVENT ORAL HEALTH PROBLEMS

Chew sugar-free gum or xylitol gum to help make teeth strong and stop decay.

Avoid tobacco and betel nut - they stain your teeth and contain ingredientsthat can lead to oral (mouth) cancer.

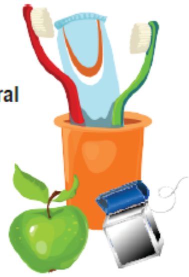

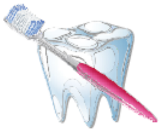

BRUSH TWICE AND FLOSS ONCE A DAY

Use a toothbrush with toothpaste to clean all parts of your teeth.

Use floss to clean between your teeth.

d. Most recent version of summary educational handout (shown here in English)

Dental Checkups: Why, What, When, and How

Going to the dentist twice a year keeps your smile bright and healthy and prevents costly health problems.

Dentists help promote

- healthy teeth and bones
- nutritional health
- a healthy heart

Dentists help prevent

- bad breath
- tooth loss
- gum disease

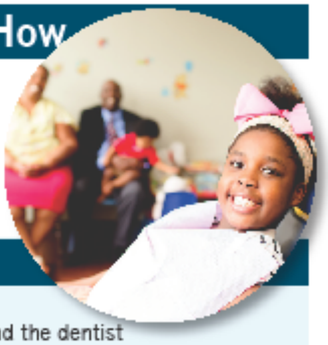

What to Expect at Your First Dental Visit

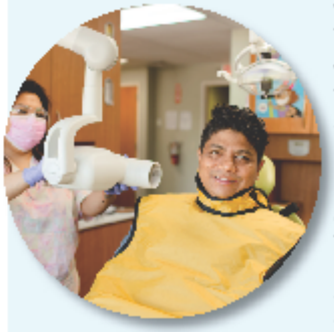

WHO WILL YOU SEE AT YOUR VISIT?
The office receptionist, then the dental hygienist and the dentist

WHAT WILL HAPPEN AT YOUR VISIT?
The dental hygienist will talk with you about your teeth and mouth (oral health) and about your general health.

X-RAYS
Pictures of your teeth and jaw will be taken while you are standing and seated.

CLEANING AND FLUORIDE
The hygienist will brush and floss your teeth, gently remove sticky plaque and bacteria with special tools, ask you to rinse your mouth with water, and may put a protective gel on your teeth for a few minutes.

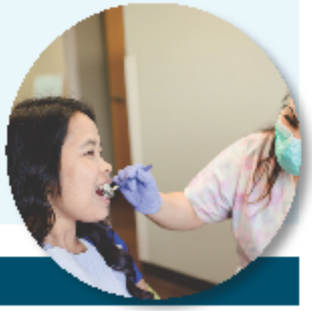

FIX AND FOLLOW-UP
The dentist will examine your teeth and fix any serious or urgent problems. The staff will discuss dental care at home and schedule your next appointment.

How to Schedule a Dental Appointment

1. Find Safety Net dental clinics here: www.oralhealthkansas.org/SafetyNet.html
2. Find a dentist that accepts KanCare here: www.oralhealthkansas.org/KanCare.html
3. Call the phone number for the clinic you select and tell scheduler if you prefer a certain day of the week, date, or time for your appointment (initial appointments last about one hour).
4. Have your calendar and any insurance cards or documents in hand during the call.

WHEN SHOULD YOU GO TO THE DENTIST?
Anytime you have an urgent dental problem, and every 6 months for a dental check-up. Please arrive 20 minutes before your appointment time. We look forward to your visit!

Dental Health at Home

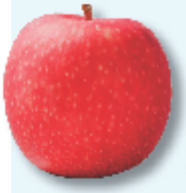

EAT HEALTHY FOOD
Avoid sugary foods and drinks. Sugar feeds germs and creates acid that harms your teeth. Crisp vegetables and fruits (like fresh carrots or apples) and calcium-rich foods (like almonds, milk or yogurt) will keep your teeth healthy and strong.

AVOID SNACKING
After eating or snacking, food and germs cause acid that attacks teeth. Snacking without brushing your teeth increases food and acid left on teeth. Three meals a day and fresh fruit or vegetable snacks, followed by brushing, reduces acid.

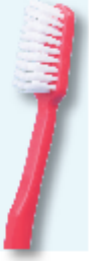

PREVENT ORAL HEALTH PROBLEMS
Chew sugar-free gum or xylitol gum to help make teeth strong and stop decay. Avoid tobacco and betel nut; they stain your teeth and contain ingredients that can lead to oral (mouth) cancer.

BRUSH TWICE A DAY AND FLOSS ONCE A DAY
Use a toothbrush with toothpaste to clean all parts of your teeth. Use floss to clean between your teeth.

e. Most recent online version of translated summary educational handouts:

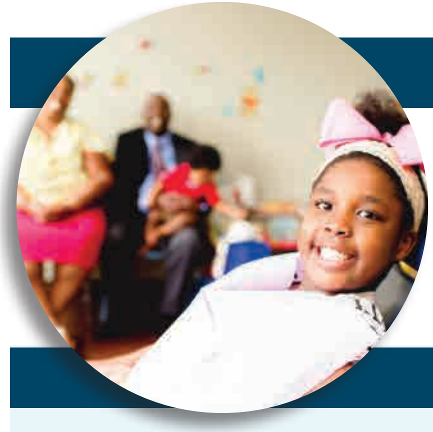

د غاښونو معاینه: ولې، کله، او څرنګه

په کال کې دوه ځله د غاښونو ډاکټر ته تلنې سره د ستاسو مسکا ځلانده او روغه وي او د ارزښتناکو روغتیایي ستونزو مخنیوی کوي.

د غاښونو ډاکټران مرسته کوي په مخنیوي کې د

- خولې د بوی
- د غاښونو لوېدنه
- د وريو ناروغی

د غاښونو ډاکټران مرسته کوي په وده کې د

- د روغو غاښونو او هډوکو
- تغذیې سره تړاو لرونکې روغتیا کې
- روغ زړه

د غاښونو د درملنې لپاره لومړنۍ لیدنه کې د څه هیله لرلای شئ

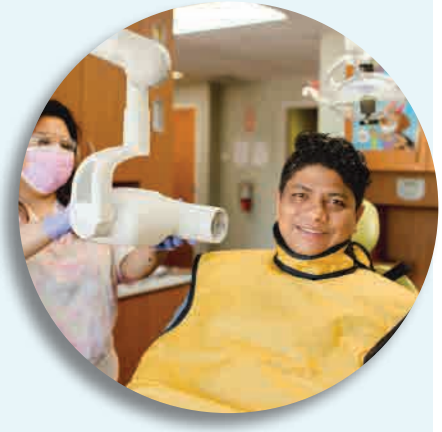

په خپله لومړنۍ لیدنه کې به چا سره وینئ؟
دفتر ته ښه راغلاست ویونکی، بیا د غاښونو د نظافت کارمند او بیا د غاښونو ډاکټر

د ستاسو په لومړنۍ لیدنه کې به کاروونه ترسره کیږي؟
د غاښونو د نظافت کارمند به تاسو سره د ستاسو د غاښونو او د خولې (شفاهي روغتیا) په اړه او د ستاسو عمومي روغتیا په اړه خبرې وکړي.

ایکسرې ګانې
په ناست او په ولاړ حالت کې به د ستاسو د غاښونو او د زامې عکسونه اخستل کیږي.

پاکونه او فلوراید
د نظافت کارمند به د ستاسو په غاښونو کې برش او د غاښونو مزی ووهي، او ځانګړو اوزارو سره به په ارام ډول داغونه او باکتیریا پاک کړي، او له تاسو به وغواړي چې خوله په اوبو کنګال کړئ، او ښایي تاسو د څو دقیقو لپاره په غاښونو ژغورونکی ملهم ولګوي.

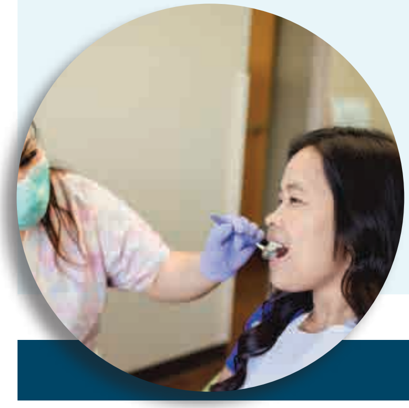

سموني او تعقیبي درملنه
د غاښونو ډاکټر به د ستاسو غاښونه معاینه کړي او کومې جدي یا عاجلې ستونزې به سمې کړي. مرستندویه کارمندان به تاسو سره په کور کې د غاښونو د خیال ساتنې په اړه خبرې درسره وکړي او د ستاسو د بلې لیدنې وخت به وټاکي.

د غاښونو د معاینې لپاره د لیدنې وخت څرنګه واخلئ

1. د Safety Net د غاښونو د درملنې کلینیکونه دلته ومومئ: www.oralhealthkansas.org/SafetyNet.html
2. داسې د غاښونو ډاکټر چې KanCare قبلوي، دلته ومومئ: www.oralhealthkansas.org/KanCare.html
3. د خوښ کړي کلینک په ټیلیفون شمېر زنګ ووهئ او د لیدنې وخت ثبتوونکي ته د لیدنې لپاره د خپلې خوښې اوونی، نېټه، یا وخت ووایئ (لومړنی لیدنې تر یو ساعت پورې اوږدې وي).
4. د زنګ په ترڅ کې خپل تقویم یعني کالیز او که د بیمې کارډونه یا اسناد لرئ ځان سره نږدې وساتئ.

تاسو باید کله د غاښونو ډاکټر ته لاړ شئ؟
کله هم چې تاسو د غاښونو عاجله ستونزه لرئ او هر 6 میاشتې وروسته د غاښونو د معاینې لپاره لاړ شئ. مهرباني وکړئ د خپلې لیدنې د وخت څخه 20 دقیقې وړاندې ځان راورسوئ. موږ تاسو سره د لیدو په هیله یو!

په کور کې د غاښونو خیال ساتنه

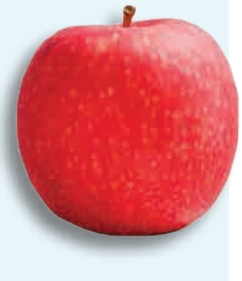

روغتیا ته ښه خواړه وخورئ
له شکرې ډکو خواړو او څښاکونو څخه ډډه وکړئ. شکره د جراثیمو خوراک دی او غاښونو ته زیانمن تېزابي موادو پیدا کوي. خوړلو کې خرپ کوونکې سبزۍ او مېوې (لکه تازه ګاځرې یا منې) او له کېلشیم ډک خوراکونه (لکه بادام، شودې یا ماسته) به د ستاسو غاښونه روغ او قوي وساتي.

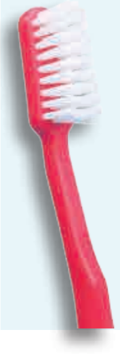

بې وخته خوراکونو څخه ډډه وکړئ
د ډوډۍ خوړلو یا بې وخته خوراک کولو وروسته، خوراک او جراثیم په غاښونو برید کوونکې تېزاب پیدا کوي. بې وخته خوراک او وريېسې غاښونه نه برش کولو له امله په غاښونو کې پاتې خوراک او تېزاب زیاتوي. په ورځ کې درې ځله ډوډۍ او تازه مېوې یا د سبزۍ سپک خوراکونه، چې خوړلو پسې یې برش وشي، تېزاب کموي.

د شفاهي روغتیا د ستونزو مخنیوی وکړئ
له شکرې پرته ژاولې یا د زایلیتول ژاولې خورئ تر څو غاښونو مو قوي شي او وروستېدنه یې ودریږي. له تماکو او پان څخه ډډه وکړئ؛ دا غاښونه داغوي او داسې مواد لري چې د شفاهي (د خولې) د کېنسر لامل کیږي.

په ورځ کې دوه ځله غاښونه برش کوي او یو ځل د غاښونو مزی وهئ
د غاښونو ټولې برخې پاکولو لپاره د غاښونو د برش د پاسه د غاښونو دوایي کاروئ. د غاښونو منځ کې پاکوالي لپاره د غاښونو مزی کاروئ.

သွားဘက်ဆိုင်ရာ စစ်ဆေးခြင်းများ: ဘာကြောင့်လဲ၊ ဘယ်တော့လဲ၊ နှင့် ဘယ်လိုလုပ်ရမလဲ

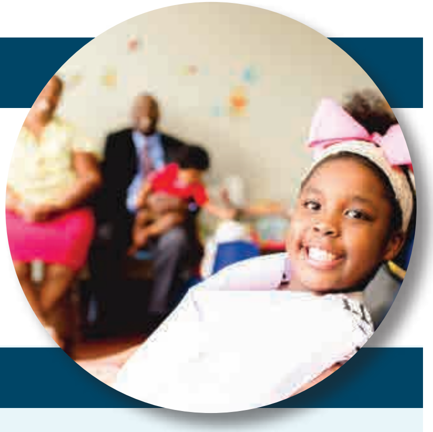

သွားဘက်ဆိုင်ရာ ဆရာဝန်ထံသို့ တစ်နှစ်လျှင် နှစ်ကြိမ်သွားခြင်းက သင့်သွားများကို ဖြူစင်ကျန်းမာစေပြီး ကုန်ကျစရိတ်များသည့် ကျန်းမာရေးပြဿနာများမှ ကာကွယ်ပေးသည်။

သွားဘက်ဆိုင်ရာဆရာဝန်များက အောက်ပါတို့ကိုကောင်းမွန်တိုးတက်စေသည်

- ကျန်းမာသော သွားများနှင့် အရိုးများ
- အာဟာရဆိုင်ရာ ကျန်းမာရေး
- ကျန်းမာသန်စွမ်းသော နှလုံး

သွားဘက်ဆိုင်ရာဆရာဝန်များက အောက်ပါတို့ကိုကာကွယ်ပေးသည်

- ခံတွင်း အနံ့ဆိုးခြင်း
- သွားဆုံးရှုံးခြင်း
- သွားဖုံးရောဂါ

သင့်ပထမဆုံး သွားဘက်ဆိုင်ရာ သွားရောက်မှုတွင် ဘာတွေလုပ်မလဲ

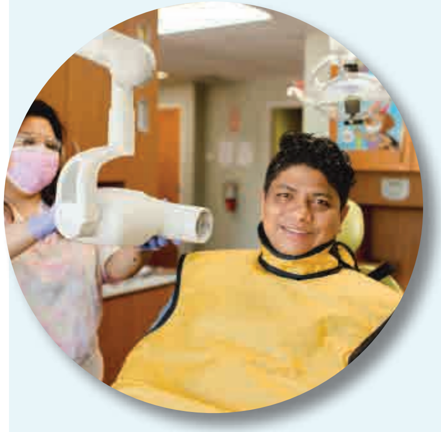

သင်သွားရောက်သောအခါ မည်သူကိုတွေ့ရမလဲ?
ရုံးခန်းခရီးဦးကြိုဆိုသူ၊ ပြီးလျှင် သွားဘက်ဆိုင်ရာ ကျန်းမာရေးပညာပေးသူ နှင့် သွားဘက်ဆိုင်ရာဆရာဝန်

သင်သွားရောက်သောအခါ ဘာတွေလုပ်မလဲ?
သွားဘက်ဆိုင်ရာ ကျန်းမာရေးပညာပေးသူက သင့်အား သင့်သွားနှင့် ခံတွင်း (ခံတွင်းကျန်းမာရေး) နှင့် သင့်အထွေထွေကျန်းမာရေးအကြောင်းကို ပြောဆိုမည်။

ဓါတ်မှန်များ
သင်ရပ်နေစဉ် နှင့် ထိုင်နေစဉ် သင့်သွားနှင့် မေးရိုးကို ဓါတ်ပုံများရိုက်မည်။

သန့်ရှင်းခြင်း နှင့် ဖလူအိုရိုက်
ပညာပေးသူက သင့်သွားများကို တိုက်ပြီး သွားသန့်စင်ကြိုးဖြင့်သန့်ရှင်းပေးမည်၊ ကပ်နေသောသွားချေးများနှင့် ဘက်တီးရီးယားကို အထူးကိရိယာများဖြင့် ငြင်သာစွာဖယ်ရှားခြင်း၊ သင့်အားရေဖြင့် ပလုပ်ကျင်းခိုင်းခြင်းပြီးလျှင် သင့်သွားများကို မိနစ်အနည်းငယ်မျှ အကာအကွယ်ပေးသည့် အဆီပျစ်ကို ထည့်ပေးကောင်းထည့်ပေးမည်။

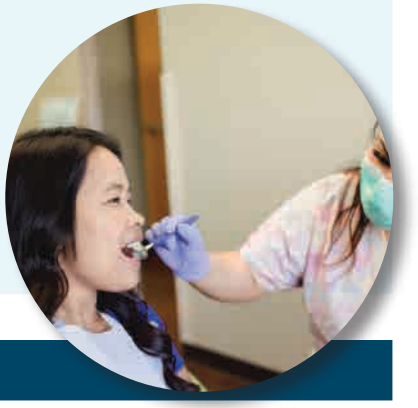

ပြုပြင်ခြင်းများ နှင့် နောက်ထပ်လုပ်ဆောင်ရန်
သွားဘက်ဆိုင်ရာဆရာဝန်က သင့်သွားများကို စစ်ဆေးပြီး ပြင်းထန်သော သို့မဟုတ်အရေးတကြီးဖြစ်နေသော ပြဿနာများကို ပြုလုပ်ပေးမည်။ ဝန်ထမ်းက အိမ်တွင် သွားဘက်ဆိုင်ရာ စောင့်ရှောက်မှုအကြောင်း ဆွေးနွေးပြီး သင့်နောက်ထပ်ချိန်းဆိုမှုကို စီစဉ်ပေးမည်။

သွားဘက်ဆိုင်ရာ ချိန်းဆိုမှုကို ဘယ်လိုလုပ်ရမလဲ

1. Safety Net သွားဘက်ဆိုင်ရာ ဆေးခန်းများကို ဤနေရာတွင်ရှာပါ: www.oralhealthkansas.org/SafetyNet.html
2. KanCare ကိုလက်ခံသော သွားဘက်ဆိုင်ရာဆရာဝန်ကို ဤနေရာတွင်ရှာပါ: www.oralhealthkansas.org/KanCare.html
3. သင်ရွေးသောဆေးခန်း၏ ဖုန်းနံပါတ်ကို ခေါ်ဆိုပြီး ချိန်းဆိုမှုလုပ်ပေးသူအား ချိန်းဆိုမှုအတွက် သင်နှစ်သက်သော ရက်သတ္တပတ်၏ အချိန်နှင့်၊ သို့မဟုတ် အချိန်ကိုပြောပါ (စစချင်းလုပ်သောချိန်းဆိုမှုများသည် တစ်နာရီခန့်ကြာနိုင်သည်)။
4. ခေါ်ဆိုမှုအတွင်း သင့်ပကြာ့ခဒိန် နှင့် အာမခံကတ်ပြားများ သို့မဟုတ် စာရွက်စာတမ်းများကို အဆင်သင့်ထားရှိပါ။

သွားဘက်ဆိုင်ရာဆရာဝန်ထံ မည်သည့်အချိန်တွင်သွားသင့်သလဲ?
သင့်ထံတွင် အရေးကြီးသော သွားဘက်ဆိုင်ရာပြဿနာ နှင့် ၆ လတကြိမ် သွားဘက်ဆိုင်ရာစစ်ဆေးမှုလုပ်ရန် ရှိပါက။ သင်၏ ချိန်းဆိုမှုအချိန်မတိုင်မီ မိနစ် ၂၀ ကြိုရောက်ပါစေ။ သင့်ကို ကျွန်ုပ်တို့စောင့်မျှော်နေပါသည်!

အိမ်တွင်း သွားကျန်းမာရေး

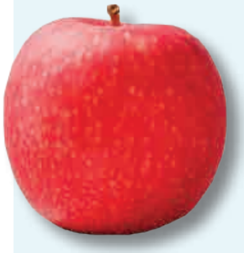

ကျန်းမာရေးနှင့် ညီညွတ်သော အစားအစာကိုစားပါ
သကြားပါသော အစာနှင့် အချိုရည်များကို ရှောင်ပါ။ သကြားသည် ပိုးများအတွက်အစာဖြစ်စေပြီး သင့် သွားများကို ထိခိုက်စေသည့် အက်စစ်ကိုထုတ်ပေးသည်။ လတ်ဆတ်သော ဟင်းသီးဟင်းရွက်များနှင့် အသီးများ (လတ်ဆတ်သော မုန်လာဥများ သို့မဟုတ် ပန်းသီးများ) နှင့် ကယ်လဆီယမ်များသော အစာများ (ဗာဒံစေ့များ၊ နွားနို့ သို့မဟုတ် ဒိန်ချဉ် ကဲ့သို့) ကသင့်သွားများကို ကျန်းမာခိုင်ခန့်စေသည်။

သွားရည်စာစားခြင်းကို ရှောင်ပါ
သွားရည်စာစားပြီးလျှင် အစာနှင့် ပိုးများသည် သွားများကို ထိခိုက်စေသော အက်စစ်ကိုဖြစ်စေသည်။ သွားရည်စာစားပြီး သွားမတိုက်ခြင်းသည် အစာနှင့် အက်ဆစ်များကို သင့်သွားများပေါ်တွင်ကျန်ရှိခြင်းပိုမိုများပြားစေသည်။ တစ်နေ့အစာ သုံးခါစားပြီး လတ်ဆတ်သော အသီး သို့မဟုတ် ဟင်းသီးဟင်းရွက်သွားရည်စာများစားပါ၊ ပြီးလျှင်သွားတိုက်ပါက အက်စစ်ကို လျော့စေသည်။

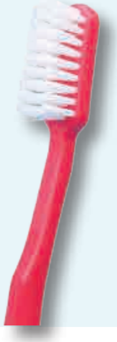

ခံတွင်းကျန်းမာရေးပြဿနာများကို ကာကွယ်ပါ
သွားများကိုခိုင်ခံ့စေရန် နှင့် သွားဆွေးမြေ့ခြင်း မဖြစ်စေရန် သကြားမပါသောဂမ်း သို့မဟုတ် ဇိုင်လီတော ဂမ်းကို ငုံပါ။ ဆေးလိပ်နှင့် ကွမ်းကိုရှောင်ပါ၊ ၎င်းတို့ကသင့်သွားများကို အရောင်စွန်းစေပြီး ခံတွင်း (ပါးစပ်) ကင်ဆာဖြစ်စေသော ပါဝင်ပစ္စည်းများ ပါရှိသည်။

သွားကို တနေ့လျှင် နှစ်ကြိမ်တိုက်ပြီး သွားသန့်စင်ကြိုးဖြင့်တစ်ကြိမ် သန့်ရှင်းပါ။
သွားပွတ်တံ နှင့် သွားတိုက်ဆေးကို အသုံးပြု၍ သင့်သွားများ၏ အစိတ်အပိုင်းအားလုံးကို သန့်ရှင်းပါ။ သင့်သွားများအကြားကို သန့်ရှင်းရန် သွားသန့်စင်ကြိုးဖြင့် သန့်ရှင်းပါ။

Baadhitaanka Ilkaha: Sababtee, Goorma, iyo Sidee

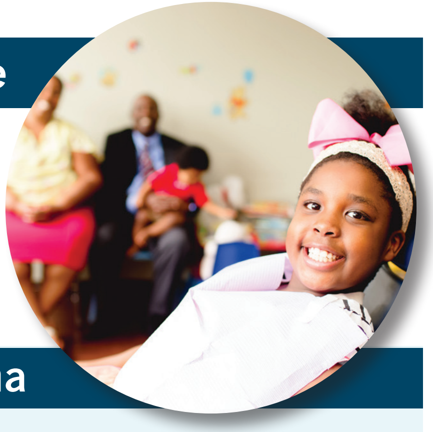

U tegida dhakhtarka ilkaha labba jeer sanadkii waxay ku ilaalisaa ilka caddayntaada mid cad oo caafimaad leh oo waxay ka hortagtaa dhibaatooyinka caafimaadka ee kharashka badan.

Dhakhtarada ilkahu waxay caawiyaan sar eu qaadista

- ilko iyo lafo caafimaad qaba
- nafaqo caafimaad leh
- wadne caafimaad qaba

Dhakhtarada ilkahu waxay ka hortagaan

- neefka xun
- ilkaha oo dhaca
- cudurada ciridka

Waxa laga filayo Booqashadaada Koowaad ee Ilkaha

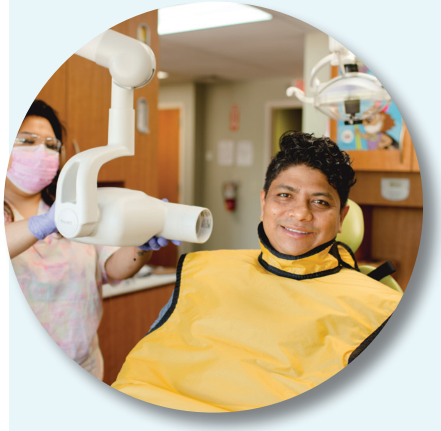

KUMAAD ARKI DOONTAA BOOQASHADAADA?
Qofka soo dhawaynta xafiiska, markaa khabiirka caafimaadka ilkaha iyo dhakhtarka ilkaha.

MAXAA DHICI DOONAA BOOQASHADAADA?
Khabiirka caafimaadka ilkahu waxa uu adiga kaala hadli doonaa ilkahaaga iyo afkaaga (caafimamadka afka) iyo wax ku saabsan caafimaadkaaga guud.

RAAJOOYINKA
Sawirada ilkahaaga iyo daanka waa la qaadi doonaa marka aad taagan tahay oo fadhido.

NADIIFINTA IYO FALOORAYDHKA
Khabiirka caafimaadka waxa uu burush marin doonaa oo dun dhexgelin doonaa ilkagaaga, si degen si uu ugaga saaro huurada ku dhegtay iyo bakteeriyada qalab gaar ah, waxa uu ku waydiin doonaa inaad biyo ku luqluqato, oo waxaa uu marin doonaa jel ilaalinaysa ilkahaaga dhowr daqiiqadood.

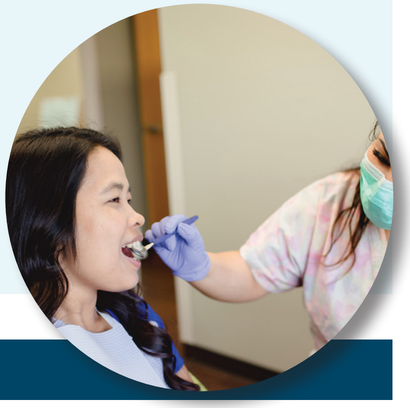

HAGAAJINTA IYO LA SOCODKEEDA
Dhakhtarka ilkahu waxa uu baadhi doonaa ilkahaaga oo waxay hagaajin doontaa dhibaatooyinka khatarta ah ama degdega ah. Shaqaalahu waxay ka hadli doonaan daryeelka ilkaha gurigaaga oo waxay kuu qaban doonaan ballanta xigta.

Sida loo qabsado Ballanta Ilakaha

1. Ka hel rugaha ilkaha Safety Net halkan www.oralhealthkansas.org/SafetyNet.html
2. Ka hel dhakhtarka ilkaha aqbala KanCare halkan: www.oralhealthkansas.org/KanCare.html
3. Soo wac lambarka telefoonka aad doroato oo u sheeg qofka qabanay ballanta haddii aad doorbidayso maalin gaar ah ama todobaad, taariikhda, ama wakhtiga ballantaada (ballamaha hore waxay gaadhaan ilaa hal saac).
4. Ku hayso jadwalkaaga taariikhda iyo kaadhadhk kasta oo caymiska ah ama dhokumentiga gacanta muddada wicitaanka.

GOORMAAD U TEGAYSAA DHAKHTARKA ILKAHA?
Wakhti kasta oo aad qabto dhibaatooyinka degdega ah ee ilkaha iyo baadhitaanka ilkaha 6 bilood oo kasta. Fadlan kaalay 20 daqiiqo ka hor wakhtiga ballantaada. Waxaanu rajaynaynaa booqashadaada!

Caafimaadka Ilkaha xaga Guriga

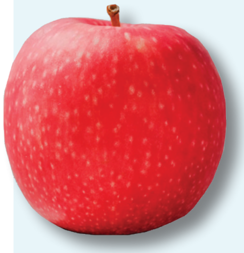

CUN CUNTADDA CAAFIMAADKA BADAN
Iska ilaali cuntadda sonkorta leh iyo cabbitaanka Sonkortu waxay quudisaa jeermiska oo waxay abuurtaa asiidh waxyeelaysa ilkahaaga. Cuntada burburta iyo khudrada (sida dabacase daama tufaac daray) iyo cuntooyinka kaalshiyamku ku badan yahay (sida almoonka, caanaha ama caaano fadhiga)waxay caafimaad ku ilaaliyaan ilkahaaga waanay xoogeeyaaan.

ISKA ILAALI CUNIDA CUNTOOYINKA FUDUD
Ka dib markaad wax cunto ama cunto fudud cunto, cunto ama jeermis ayaa sababa asiidh weerarta ilkaha. Cunuda cuntadda addigoon caddayin ilkahaaga waxya kordhisaa cuntada iyo asiidh inay ku hadho ilkaha. Saddex cunto maalintii iyo khudrad daray ah ama cuntooyin khudaar ah, oo uu ka dambeeyo caddaygu, waxay yareeyaan asiidha.

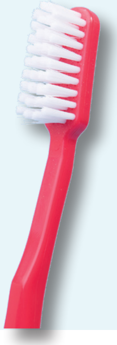

KA HORTAG DHIBAATOOYINKA CAAFIMAADKA AFKA
Calaasho xanjada aan sonkorta lahayn ama xanjada xyltol si au caawiso inay ilkaha xoogayso oo ka joojiso suuska. Iska ilaali buuriga ama midhaha betalka; waxay ku reebi karaan midab wasakh ah ilkahaaga oo waxay ka kooban yihiin waxyaabo keeni kara kansarka (afka).

LABBA GOOR CADDAY MAALINTII OO DUN KU NADIIFI ILKAHA HAL MAR MAALIINTII
Ku isticmaal burushka caddayga cajiinka ilkaha si aad u nadiifiso dhammaan qaybaha ilkahaaga. Isticmaalka dunta nadiifinta si aad u nadiifiso ilkahaaga dhexdooda.

فحوصات الأسنان: سبب إجرائها والمواعيد وكيفية إجرائها

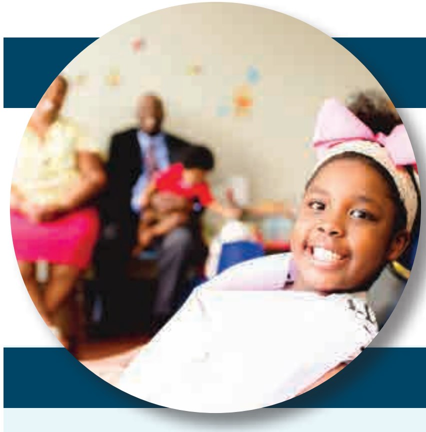

زيارة طبيب الأسنان مرتين في السنة يحافظ على ابتسامتك مشرقة وصحية وتقي من التعرض لمشاكل صحية مكلفة.

يساعد أطباء الأسنان في الوقاية من

- رائحة الفم السيئة
- فقدان الأسنان
- أمراض اللثة

يساعد أطباء الأسنان في تعزيز

- صحة الأسنان والعظام
- الصحة الغذائية
- صحة القلب

ماذا الذي تتوقع حدوثه في أول زيارة لك لطبيب الأسنان؟

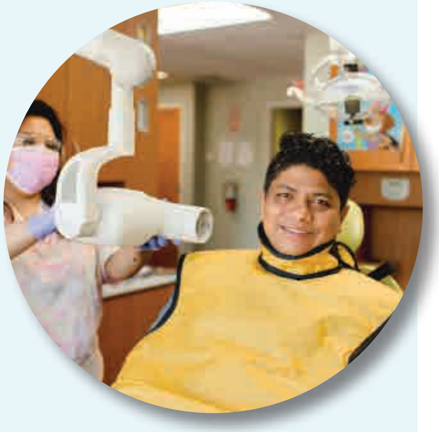

من الذي ستقابله في زيارتك؟

موظف الاستقبال ثم أخصائي صحة الأسنان وطبيب الأسنان

ما الذي سيحدث في هذه الزيارة؟

سيتناقش أخصائي صحة الأسنان معك حول أسنانك وفمك (صحة الفم) وحول صحتك العامة.

الأشعة السينية

سيتم التقاط صور لأسنانك وفكك وأنت في وضعية الوقوف والجلوس.

التنظيف والفلوريد

سيقوم أخصائي صحة الأسنان بتنظيف أسنانك بالفرشاة والخيط وإزالة الجير والبكتيريا المتراكمة على أسنانك برفق باستخدام أدوات خاص وسيطلب منك شطف فمك بالماء وقد يضع جلا واقيًا على أسنانك لبضع دقائق.

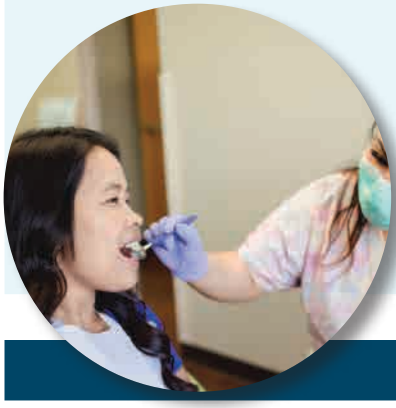

المعالجة والمتابعة

سيفحص طبيب الأسنان أسنانك وسيعالج أي مشاكل خطيرة أو عاجلة. سيتناقش الموظف معك حول رعاية الأسنان في المنزل وسيحدد موعد زيارتك التالية.

كيفية تحديد موعد لزيارة طبيب الأسنان

1. يمكنك العثور على عيادات الأسنان التابعة لشبكة الأمان (Safety Net) من خلال الموقع الإلكتروني التالي: www.oralhealthkansas.org/SafetyNet.html
2. يمكنك البحث عن طبيب أسنان يقبل برنامج كانساس للرعاية الصحية (KanCare) هنا: www.oralhealthkansas.org/KanCare.html
3. اتصل برقم هاتف العيادة التي تختارها وأخبر المسؤول عن تنظيم المواعيد إذا كنت تفضل الحضور في يوم أو تاريخ أو موعد معين (المواعيد الأولية تستغرق حوالي ساعة واحدة).
4. اجعل الموعد الذي اخترته وأي بطاقات تأمينية أو وثائق في متناول يديك أثناء المكالمة.

متى يجب عليك زيارة طبيب الأسنان؟

في أي وقت تعاني فيه من مشكلة طارئة في الأسنان وتحتاج إلى الخضوع إلى فحص أسنان كل 6 أشهر. يُرجى الوصول قبل 20 دقيقة من موعدك. نحن نتطلع إلى زيارتك لنا

العناية بصحة الأسنان في المنزل

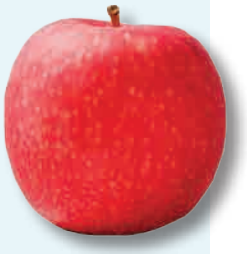

تناول طعامًا صحيًا

تجنب تناول الأطعمة والمشروبات الغنية بالسكريات حيث أن السكر يغذي الجراثيم ويكون حمضًا يضر أسنانك. الخضار والفواكه المقرمشة (مثل الجزر أو التفاح الطازج) والأطعمة الغنية بالكالسيوم (مثل اللوز أو الحليب أو الزبادي) ستحافظ على صحة أسنانك وقوتها.

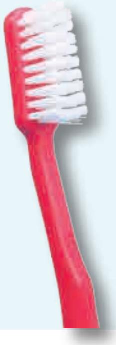

تجنب تناول الوجبات الخفيفة

بعد تناول الأكل أو الوجبات الخفيفة يتسبب الطعام والجراثيم في تكوين حمضًا يهاجم الأسنان. تناول الوجبات الخفيفة دون غسل الأسنان بالفرشاة من شأنه أن يزيد من كمية الطعام والحمض المتراكمة على الأسنان. تناول ثلاث وجبات في اليوم وفاكهة طازجة أو وجبات خفيفة نباتية وغسل الأسنان بعد أي من ذلك من شأنه تقليل كمية الحمض.

الوقاية من مشاكل صحة الفم

امضغ علكة خالية من السكر أو علكة زيليتول للمساعدة في تقوية الأسنان وإيقاف التسوس. تجنب استخدام التبغ وجوز التنبول حيث أنها تؤدي إلى تصبغ الأسنان وتحتوي على مكونات يمكن أن تؤدي إلى الإصابة بسرطان الفم.

اغسل أسنانك بالفرشاة مرتين يوميًا واستخدم الخيط مرة واحدة يوميًا

اغسل أسنانك بالفرشاة باستخدام معجون أسنان لتنظيف جميع أجزاء أسنانك. استخدم الخيط لتنظيف الفراغات الموجودة بين أسنانك.

Chequeos odontológicos: por qué, cuándo y cómo

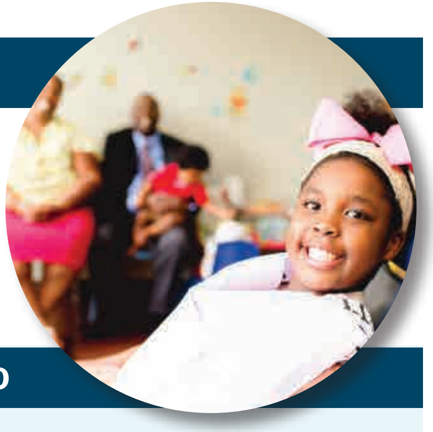

Ir al odontólogo dos veces al año mantiene su sonrisa brillante y saludable, y previene problemas de salud costosos.

Los odontólogos ayudan a promover lo siguiente
- dientes y huesos sanos;
- la salud nutricional;
- un corazón sano.

Los odontólogos ayudan a prevenir lo siguiente
- el mal aliento;
- la pérdida de dientes;
- las enfermedades en las encías.

Qué esperar en su primera consulta con el odontólogo

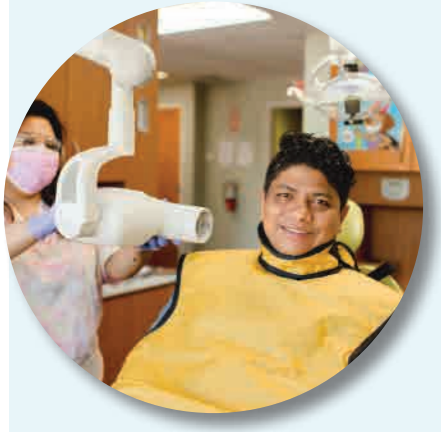

¿A QUIÉN VERÁ EN SU CONSULTA?
Al recepcionista del consultorio, luego al higienista bucal y, por último, al odontólogo.

¿QUÉ SUCEDERÁ EN SU CONSULTA?
El higienista bucal hablará con usted sobre los dientes y la boca (salud bucal), así como sobre su salud general.

RADIOGRAFÍAS
Se le tomarán imágenes de los dientes y la mandíbula mientras está de pie y sentado.

LIMPIEZA Y FLÚOR
El higienista le cepillará los dientes y usará hilo dental, eliminará suavemente la placa pegajosa y las bacterias con herramientas especiales, le pedirá que se enjuague la boca con agua y puede ponerle un gel protector en los dientes durante unos minutos.

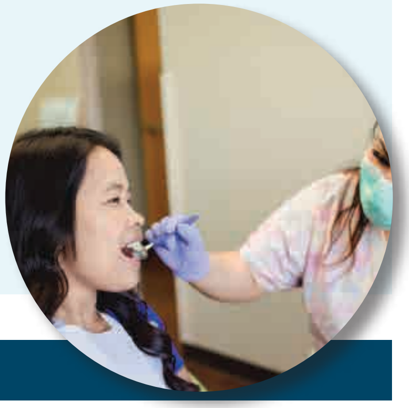

ARREGLOS Y SEGUIMIENTO
El odontólogo le examinará los dientes y arreglará los problemas graves o urgentes. El personal le explicará el cuidado dental que debe tener en el hogar y programará su próxima cita.

Cómo programar una cita odontológica

1. Encuentre las clínicas odontológicas de Safety Net aquí: www.oralhealthkansas.org/SafetyNet.html
2. Encuentre un odontólogo que acepte KanCare aquí: www.oralhealthkansas.org/KanCare.html
3. Llame al número de teléfono de la clínica que haya seleccionado e informe a quien programa las consultas si prefiere un determinado día de la semana, fecha u hora para su cita (las citas iniciales duran una hora, aproximadamente).
4. Tenga su calendario y la tarjeta del seguro médico o los documentos correspondientes a mano durante la llamada.

¿CUÁNDO DEBE IR AL ODONTÓLOGO?
Toda vez que tenga un problema dental urgente y para un chequeo odontológico cada seis meses. Tenga a bien llegar veinte minutos antes de la hora de su cita. ¡Esperamos su consulta!

Salud dental en el hogar

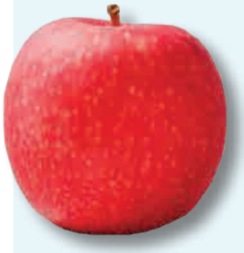

COMA ALIMENTOS SALUDABLES
Evite los alimentos y las bebidas azucarados. El azúcar alimenta los gérmenes y crea un ácido que daña los dientes. Las verduras y las frutas crujientes (como zanahorias o manzanas frescas) y los alimentos ricos en calcio (como almendras, leche o yogur) mantendrán los dientes sanos y fuertes.

EVITE LOS TENTEMPIÉS
Después de ingerir comidas o tentempiés, los alimentos y los gérmenes generan un ácido que ataca los dientes. Comer tentempiés sin cepillarse los dientes aumenta los restos de alimento y de ácido que queda en los dientes. Tres comidas al día y frutas o verduras frescas como tentempiés, seguidos de un cepillado, reducen el ácido.

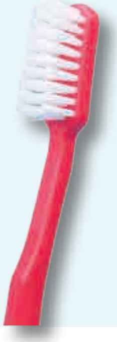

CÓMO PREVENIR LOS PROBLEMAS DE SALUD BUCAL
Consuma goma de mascar sin azúcar o goma de xilitol para fortalecer los dientes y detener la caries. Evite el tabaco y la nuez de betel porque manchan los dientes y contienen ingredientes que pueden provocar cáncer bucal (de boca).

CEPÍLLESE LOS DIENTES DOS VECES AL DÍA Y USE HILO DENTAL UNA VEZ AL DÍA
Use un cepillo con pasta de dientes para limpiar todas las partes de los dientes. Use hilo dental para limpiar entre los dientes.

दन्त परीक्षण: किन, कहिले र कसरी

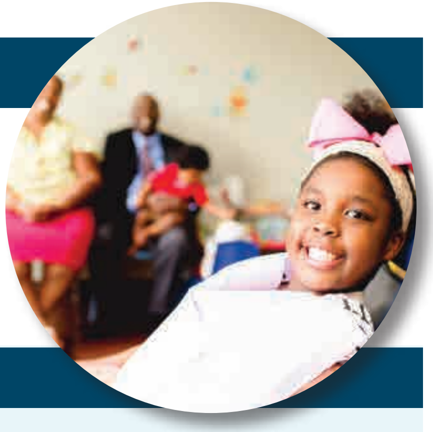

तपाईंको दन्त विशेषज्ञलाई वर्षको दुई पटक भेट्नाले तपाईंको मुस्कानमा चमक र स्वस्थता प्रदान गर्छ र महंगा स्वास्थ्य समस्याहरूबाट रोकथाम गर्छ।

दन्त विशेषज्ञले निम्न प्रवर्द्धनमा मद्दत गर्नुहुन्छ

- स्वस्थ दाँत र हड्डी
- पोषणयुक्त स्वास्थ्य
- स्वस्थ मुटु

दन्त विशेषज्ञले निम्न रोकथाममा मद्दत गर्नुहुन्छ

- मुखको खराब गन्ध
- दाँत झर्ने
- गिंजाको रोग

तपाईंको पहिलो दन्त भेटमा के अपेक्षा गर्ने

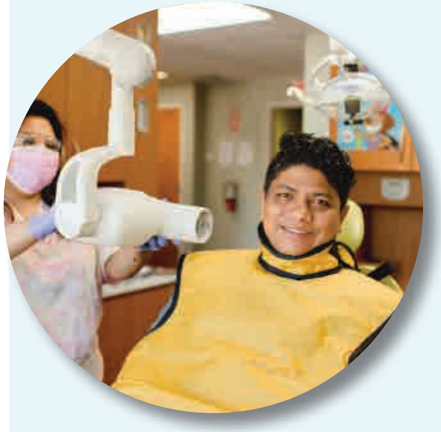

तपाईंले आफ्नो भेटमा कसलाई भेट्नुहुनेछ?
कार्यालयको रिसेप्शनमा बस्ने व्यक्ति, त्यसपछि दन्त सरसफाइ विशेषज्ञ र दन्त चिकित्सक

तपाईंको पहिलो भेटमा के हुनेछ?
दन्त सरसफाइ विशेषज्ञले तपाईंको दाँत र मुख (मुखको स्वास्थ्य) बारे र तपाईंको सामान्य स्वास्थ्य बारे तपाईंसँग कुरा गर्नुहुनेछ।

एक्स-रे
तपाईं उभिएको र बसेको अवस्थामा तपाईंको दाँत र बंगाराको तस्वीर खिचिनेछ।

सरसफाइ र फ्लोराइड
दन्त सरसफाइ विशेषज्ञले तपाईंको दाँत माझिदिने र कुल्ला गरिदिनुहुनेछ, चिप्लो खिया र कीटाणुलाई विशेष औजारको प्रयोग गरी बिस्तारै हटाउनुहुनेछ, पानीले मुख धुन पठाउनुहुनेछ र केही मिनेटका लागि तपाईंको दाँतमा सुरक्षात्मक जेल लगाइदिन सक्नुहुनेछ।

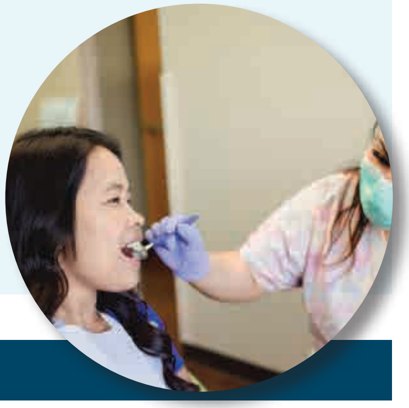

फिक्स-अप र फलो-अप
दन्त चिकित्सकले तपाईंको दाँत जाँच्नुहुनेछ र कुनै पनि गम्भीर वा आकस्मिक समस्याहरूको समाधान गर्नुहुनेछ। कर्मचारीले घरमा दाँतको स्याहार कसरी गर्ने भनी छलफल गर्नुहुनेछ र तपाईंलाई अर्को अपोइन्टमेन्ट दिनुहुनेछ।

दन्त अपोइन्टमेन्ट कसरी निर्धारण गर्ने

1. सुरक्षा नेट दन्त क्लिनिकहरू यहाँ फेला पार्नुहोस्: www.oralhealthkansas.org/SafetyNet.html
2. KanCare स्वीकार गर्ने दन्त चिकित्सक यहाँ फेला पार्नुहोस्: www.oralhealthkansas.org/KanCare.html
3. तपाईंले चयन गर्नुभएको क्लिनिकको फोन नम्बरमा फोन गर्नुहोस् र तपाईंले आफ्नो अपोइन्टमेन्टका लागि आफ्नो प्राथमिकतामा रहेको दिन, मिति वा समय बारे अपोइन्टमेन्ट मिलाउने व्यक्तिलाई बताउनुहोस् (प्राथमिक अपोइन्टमेन्टहरू करिब एक घण्टाको हुन्छन्)।
4. फोन गरिरहेको समयमा आफ्नो साथमा पात्रो र कुनै बीमा कार्ड वा कागजात राख्नुहोस्।

तपाईं दन्त चिकित्सकलाई कहिले भेट्न जानुपर्छ?
तपाईंलाई दाँतको आकस्मिक समस्या भएको कुनै पनि समयमा र हरेक 6 महिनामा दाँतको परीक्षणका लागि। कृपया तपाईंको अपोइन्टमेन्ट समयभन्दा 20 मिनेट पहिले आउनुहोला। हामी तपाईंलाई दन्त भ्रमणमा भेट्ने आशा राख्छौं!

घरमा दाँतको स्वास्थ्य

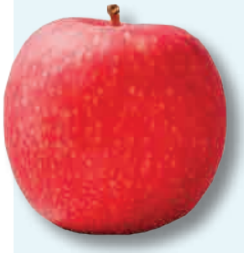

स्वस्थ खाना खानुहोस्
गुलिया खानेकुरा र पेय पदार्थ नखानुहोस्। गुलियोले कीटाणुलाई खाना उपलब्ध गराउँछ र तपाईंको दाँतलाई क्षति पुर्‍याउने अम्ल बनाउँछ। रसिला तरकारी तथा फलफूल (जस्तै, ताजा गाजर वा स्याउ) र क्याल्सियमयुक्त खानेकुरा (जस्तै, बदाम, दूध वा दही) ले तपाईंको दाँतलाई स्वस्थ र बलियो राख्नेछन्।

स्न्याकहरू नखानुहोस्
खाना वा स्न्याक खाएपछि, खाना र कीटाणुहरूले अम्ल निकाल्छन् जसले दाँतलाई आक्रमण गर्छ। स्न्याकपछि दाँत नमाझेमा यसले तपाईंको दाँतमा खाना र अम्लको मात्रा बढाउँछ। दिनमा तीन पटक खाना र स्न्याकमा ताजा फलफूल वा तरकारी खाएपछि दाँत माझेमा यसले अम्लको मात्रा कम गराउँछ।

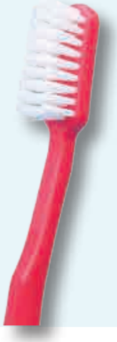

मुखको स्वास्थ्य सम्बन्धी समस्याहरूको रोकथाम गर्नुहोस्
दाँतलाई बलियो बनाउन र किराले खानबाट रोक्न चिनीरहित गम वा जाइलिटल गम चपाउनुहोस्। सुर्तीजन्य पदार्थ र सुपारी नखानुहोस्; तिनीहरूले तपाईंको दाँतमा खिया पार्छन् र तिनीहरूमा मुखको क्यान्सर गराउन सक्ने पदार्थहरू समावेश हुन्छन्।

दिनमा दुई पटक दाँत माझ्नुहोस् र दिनमा एक पटक फ्लस गर्नुहोस्
तपाईंको दाँतको सबै भागलाई सफा गर्न दाँत माझ्ने ब्रसमा दाँत माझ्ने पेस्टको प्रयोग गर्नुहोस्। तपाईंको दाँतको बीचमा सफा गर्न फ्लसको प्रयोग गर्नुहोस्।

Kungalia meno: Kwa nini, Wakati Gani na Namna Gani

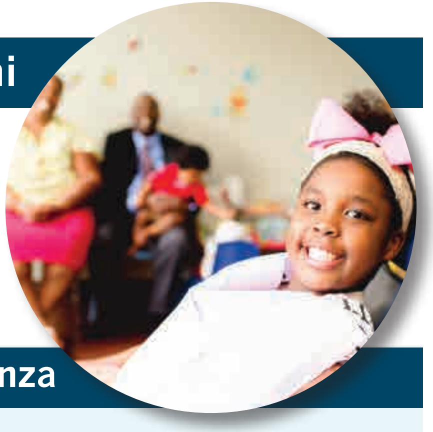

Kuenda kwa munganga wa meno mara mbili kwa mwaka inachunga kucheka kwako kungaa na kwa afya nzuri na kuzuiza matunzo ya beyi kali.

Wanganga wa meno wanasaidia kuendelesha

- meno na mifupa zinazokuwa na afya nzuri
- malisho ya afya nzuri
- moho ya afya nzuri

Wanganga wa meno wanasaidia kuzuiza

- hewa mbaya
- kupoteza meno
- ugonjwa ya fasi meno zimengiae

Nini kutarajia unapoona munganga wa meno mara ya kwanza

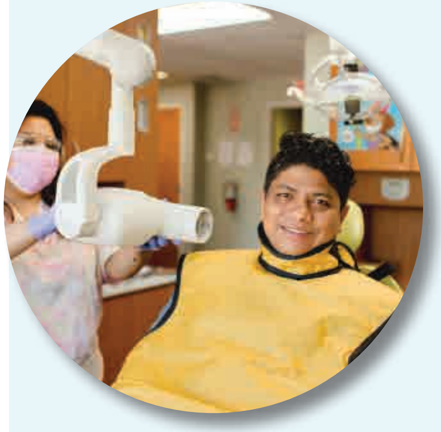

UTAONA NANI KWA MARA YA KWANZA?
Mwenye kukaribisha na kisha mwenye kufanya usafi wa meno na munganga wa meno

NINI ITAFANYIKA?
Mwenye kuangalia usafi wa meno atazungumuza nawe kuhusu meno na kinywa yako (usafi wa midomo) na kuhusu afya yako kwa jumla.

X-RAYS
Picha za meno yako na taya (machoires) zitakamatwa wakati unaposimama na unapoketi.

KUSAFISHA NA DAWA (FLUORIDE)
Mwenye kufanya usafi atasafisha na kupitisha uzi katikati ya meno yako, polepole akitosha uchafu ulikiokwama na vidudu akitumia vyombo vya kufaa, akikuuliza kusafisha kinywa chako na maji na anawezaweka mafuta ya kulinda kwa meno yako kwa dakika chache.

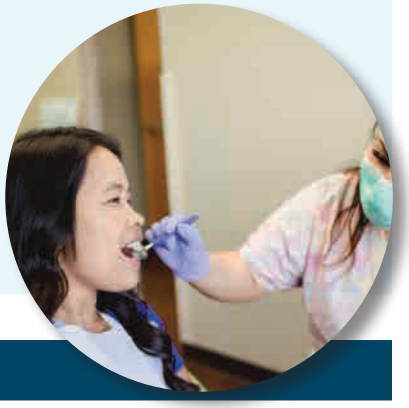

KUTENGENEZA NA KUFUATILIA
Munganga wa meno atachunguza meno yako na kutengeneza mambo yote ya muhimu na ya haraka. Wafanya kazi watazungumuzia nini kufanya kwa matunzo ya meno nyumbani na kupanga wakati gani utakuja tena.

Namna Gani Kupanga Wakati wa Kuja Kuona Munganga wa Meno

1. Pata hapa fasi nzuri kwa matunzo ya meno: www.oralhealthkansas.org/SafetyNet.html
2. Pata hapa munganga wa meno mwenye anakubal KanCare: www.oralhealthkansas.org/KanCare.html
3. Ita namba ya simu ya pahali ya matunzo uliyochagua na sema kwa mwenye kupanga kama unataka siku fulani ya juma, tarehe ao wakati kwa kufika kwako (kutayarishwa kufika mbele inadumu kama saa moja).
4. Uwe na kelnda yako na karte yeyote ya kusaidiwa (assurance) ao barua kwa mukono wakati unapoita.

UTAENDA KWA MUNGANGA WA MENO WAKATI GANI?
Wakati wowote unapokuwa na jambo ya haraka ya meno na kuangalia meno kila miezi 6. Tafazali, kuja dakika 20 mbele ya wakati wako ya kuangaliwa. Tunangojea kufika kwako!

Matunzo ya Meno Nyumbani

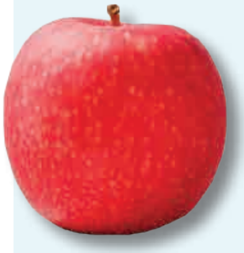

KULA CHAKULA YA KULETA AFYA NZURI
Ujizuize na vyakula na vinywaji ya sukari mingi. Sukari unakulisha vidudu na inaumba acide inayoumiza meno. Mamboga na matunda (kama karoti ao pome) na vyakula vinavyokuwa na calcium mingi (kama matunda, maziwa ao yugur) zitalinda meno kuwa na afya nzuri na nguvu.

UJIZUIZE NA VYAKULA VYEPESI (COCK-TAIL)
Kisha kukula, chakula na vidudu vinaleta acide inayoshambulia meno. Kukula vyakula ya haraka pasipo kusafisha meno inaongeza chakula na acide vilivyoachwa juu ya meno. Kukula mara tatu kwa siku na kinywaji ya matunda ao chakula ya haraka ya mboga, kufuatiwa n akusafisha meno, inapunguza acide.

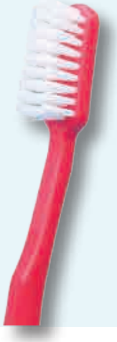

KUZUIZA SHIDA YA USAFI YA KINYWA
Kulakula vitu visivyokuwa na sukari ao xylitol kusaida kuleta nguvu kwa meno na ziache kuharibika. Ujizuize na tombako na mbegu ya beteli; vinaweka mafuta kwa meno yako na vina vitu vile vinawezapeleka kwa cancer ya kinywa.

SAFISHA MARA MBILI KWA SIKU NA PITISHA UZI MARA NOJA KWA SIKU
Tumia muswaki wa meno na dawa ya meno kwa kusafisha sehemu zote ya meno yako. Tumia uzi kwa kusafisha katikai ya meno.

Dental Checkups: Why, When, and How

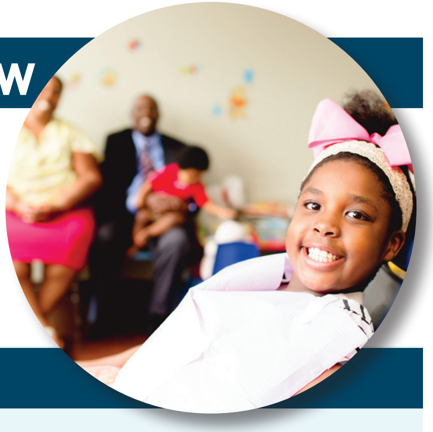

Going to the dentist twice a year keeps your smile bright and healthy and prevents costly health problems.

Dentists help promote
- healthy teeth and bones
- nutritional health
- a healthy heart

Dentists help prevent
- bad breath
- tooth loss
- gum disease

What to Expect at Your First Dental Visit

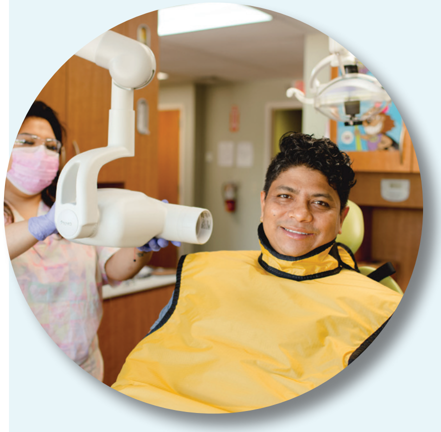

WHO WILL YOU SEE AT YOUR VISIT?
The office receptionist, then the dental hygienist and the dentist

WHAT WILL HAPPEN AT YOUR VISIT?
The dental hygienist will talk with you about your teeth and mouth (oral health) and about your general health.

X-RAYS
Pictures of your teeth and jaw will be taken while you are standing and seated.

CLEANING AND FLUORIDE
The hygienist will brush and floss your teeth, gently remove sticky plaque and bacteria with special tools, ask you to rinse your mouth with water, and may put a protective gel on your teeth for a few minutes.

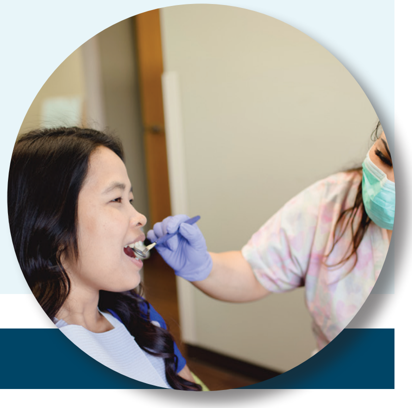

FIX-UPS AND FOLLOW-UP
The dentist will examine your teeth and fix any serious or urgent problems. The staff will discuss dental care at home and schedule your next appointment.

How to Schedule a Dental Appointment

1. Call 913-342-2552 then dial 1
2. Tell scheduler if you prefer a certain day of the week, date, or time for your appointment (initial appointments last about one hour).
3. Have your calendar and any insurance cards or documents in hand during the call.

WHEN SHOULD YOU GO TO THE DENTIST?
Anytime you have an urgent dental problem and for a dental check-up every 6 months. Please arrive 20 minutes before your appointment time. We look forward to your visit!

Dental Health at Home

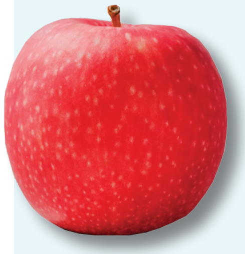

EAT HEALTHY FOOD
Avoid sugary foods and drinks. Sugar feeds germs and creates acid that harms your teeth. Crisp vegetables and fruits (like fresh carrots or apples) and calcium-rich foods (like almonds, milk or yogurt) will keep your teeth healthy and strong.

AVOID SNACKING
After eating or snacking, food and germs cause acid that attacks teeth. Snacking without brushing your teeth increases food and acid left on teeth. Three meals a day and fresh fruit or vegetable snacks, followed by brushing, reduces acid.

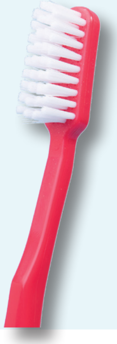

PREVENT ORAL HEALTH PROBLEMS
Chew sugar-free gum or xylitol gum to help make teeth strong and stop decay. Avoid tobacco and betel nut; they stain your teeth and contain ingredients that can lead to oral (mouth) cancer.

BRUSH TWICE A DAY AND FLOSS ONCE A DAY
Use a toothbrush with toothpaste to clean all parts of your teeth. Use floss to clean between your teeth.

Estimated cost depends on insurance type or household income. Vibrant Health can create a personal payment schedule for you. **For more information call 913-342-2552 then dial 1**

Dental Checkups: Why, When, and How

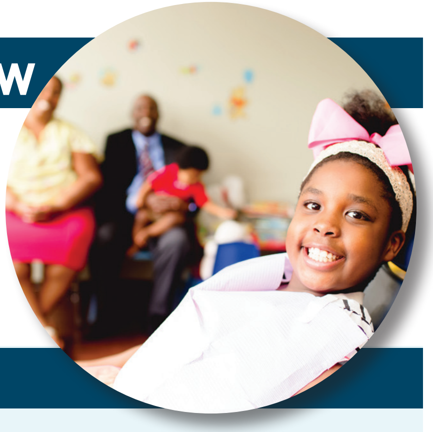

Going to the dentist twice a year keeps your smile bright and healthy and prevents costly health problems.

Dentists help promote

- healthy teeth and bones
- nutritional health
- a healthy heart

Dentists help prevent

- bad breath
- tooth loss
- gum disease

What to Expect at Your First Dental Visit

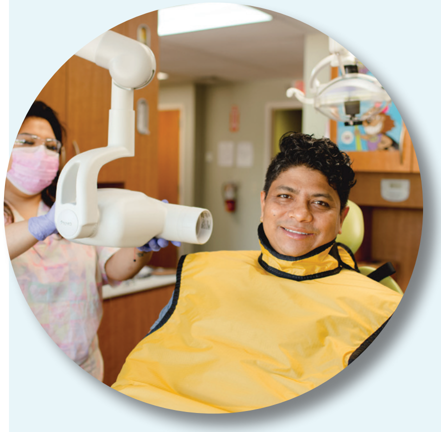

WHO WILL YOU SEE AT YOUR VISIT?
The office receptionist, then the dental hygienist and the dentist

WHAT WILL HAPPEN AT YOUR VISIT?
The dental hygienist will talk with you about your teeth and mouth (oral health) and about your general health.

X-RAYS
Pictures of your teeth and jaw will be taken while you are standing and seated.

CLEANING AND FLUORIDE
The hygienist will brush and floss your teeth, gently remove sticky plaque and bacteria with special tools, ask you to rinse your mouth with water, and may put a protective gel on your teeth for a few minutes.

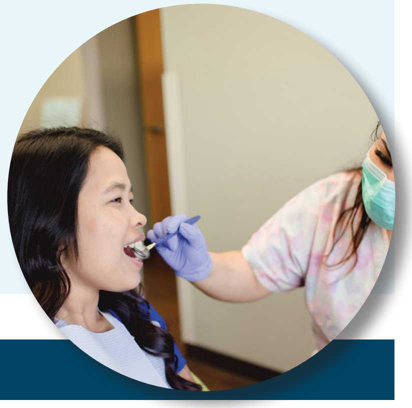

FIXES AND FOLLOW-UP
The dentist will examine your teeth and fix any serious or urgent problems. The staff will discuss dental care at home and schedule your next appointment.

How to Schedule a Dental Appointment

1. Find Safety Net dental clinics here: www.oralhealthkansas.org/SafetyNet.html
2. Find a dentist that accepts KanCare here: www.oralhealthkansas.org/KanCare.html
3. Call the phone number for the clinic you select and tell scheduler if you prefer a certain day of the week, date, or time for your appointment (initial appointments last about one hour).
4. Have your calendar and any insurance cards or documents in hand during the call.

WHEN SHOULD YOU GO TO THE DENTIST?
Anytime you have an urgent dental problem and for a dental check-up every 6 months. Please arrive 20 minutes before your appointment time. We look forward to your visit!

Dental Health at Home

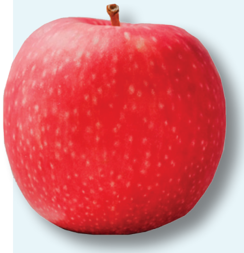

EAT HEALTHY FOOD
Avoid sugary foods and drinks. Sugar feeds germs and creates acid that harms your teeth. Crisp vegetables and fruits (like fresh carrots or apples) and calcium-rich foods (like almonds, milk or yogurt) will keep your teeth healthy and strong.

AVOID SNACKING
After eating or snacking, food and germs cause acid that attacks teeth. Snacking without brushing your teeth increases food and acid left on teeth. Three meals a day and fresh fruit or vegetable snacks, followed by brushing, reduces acid.

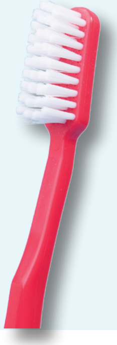

PREVENT ORAL HEALTH PROBLEMS
Chew sugar-free gum or xylitol gum to help make teeth strong and stop decay. Avoid tobacco and betel nut; they stain your teeth and contain ingredients that can lead to oral (mouth) cancer.

BRUSH TWICE A DAY AND FLOSS ONCE A DAY
Use a toothbrush with toothpaste to clean all parts of your teeth. Use floss to clean between your teeth.

Appendix 2: Final Survey Instrument

Survey: Refugee Knowledge, Attitudes, Beliefs, & Behaviors Towards Dental Health

Health care workers at the University of Kansas Medical Center (KUMC) want to understand what you and other people in your refugee community know about dental health and how you feel about the health of your teeth and gums. Participation involves completing a survey then watching a short video about healthy teeth and gums. We will ask you to complete another survey in about 8 weeks. Completing the surveys and watching the video is voluntary (you do not have to answer the questions if you do not want to) and will not affect any care or services you receive. If you answer the questions, we hope we can learn what we can do to help you and other people in your refugee community to have healthy teeth and gums. If you have any questions, please contact Dr. Kelly Kreisler at 913-588-6300. For questions about the rights of research participants, you may contact the KUMC Institutional Review Board (IRB) at (913) 588-1240 or IRBhelp@kumc.edu. Thank you.

ADMINISTRATOR ONLY: Subject ID ___________ Check one: Pre-Survey _____ Post-Survey _____

1. How old are you?	___________ years old
2. What language do you prefer to speak?	____________________
3. How many months or years have you lived in the United States of America (USA)?	___________ months
4. Do you have dental health insurance?	0= No<br>1= Yes<br>x= I don't know
5. Do you receive appropriate dental care?	0= No<br>1= Yes<br>x= I don't know
6. It is recommended that you brush your teeth how many times a day?	0= It is not necessary to brush teeth each day<br>1= 1 time each day<br>2= 2 times each day<br>3= 3 times each day<br>4= After each meal and after each snack<br>x= I don't know
7. It is recommended that you floss your teeth how many times a day?	0= It is not necessary to floss teeth each day<br>1= 1 time each day<br>2= 2 times each day<br>3= 3 times each day<br>4= After each meal and after each snack<br>x= I don't know
8. It is recommended that you visit the dentist at least how many times a year?	0= It is not necessary to visit the dentist each year<br>1= 1 time each year<br>2= 2 times each year<br>3= 3 times each year<br>4= 12 times each year<br>x= I don't know

9. How often do you brush your teeth?	0= Less than once a day<br>1= 1 time a day<br>2= 2 times a day<br>3= 3 times a day<br>4= 4 times a day or more, or after each meal & snack
10. How often do you floss your teeth?	0= Less than once a day<br>1= 1 time a day<br>2= 2 times a day<br>3= 3 times a day<br>4= 4 times a day or more, or after each meal & snack
11. How important do you consider your dental health compared to your overall health?	1= Much less important<br>2= Less important<br>3= About the same importance<br>4= More important<br>5= Much more important
12. Where do you go when you have problems with your teeth?	1= Dentist /dentist office<br>2= Doctor/doctor's office<br>3= Pharmacist /pharmacy<br>0= No preference<br>x= Other (fill in the blank): ______________________________
13. How many months has it been since your last dental visit?	_____ months<br>n= never been to the dentist
14. How many months has it been since you last *scheduled* a dental visit (for yourself)?	_____ months<br>n= never scheduled a dentist visit<br>x = do not know how to schedule
